# Alveolar cell fate selection and lifelong maintenance of AT2 cells by FGF signaling

**DOI:** 10.1038/s41467-022-34059-1

**Published:** 2022-11-21

**Authors:** Douglas G. Brownfield, Alex Diaz de Arce, Elisa Ghelfi, Astrid Gillich, Tushar J. Desai, Mark A. Krasnow

**Affiliations:** 1 Department of Biochemistry and Howard Hughes Medical Institute, Stanford University School of Medicine, Stanford, CA 94305-5307 USA; 2 Molecular and Integrative Physiological Sciences Program, Harvard T.H. Chan School of Public Health, Boston, MA USA; 3 Department of Internal Medicine and Stem Cell Institute, Stanford University School of Medicine, Stanford, CA 94305 USA; 4 Present Address: Division of Pulmonary and Critical Care Medicine, Departments of Physiology and Biomedical Engineering and of Biochemistry and Molecular Biology, Mayo Clinic College of Medicine and Science, Rochester, MN 55905 USA

**Keywords:** Differentiation, Cell lineage, Cell signalling, Self-renewal

## Abstract

The lung’s gas exchange surface is comprised of alveolar AT1 and AT2 cells that are corrupted in several common and deadly diseases. They arise from a bipotent progenitor whose differentiation is thought to be dictated by differential mechanical forces. Here we show the critical determinant is FGF signaling. Fgfr2 is expressed in the developing progenitors in mouse then restricts to nascent AT2 cells and remains on throughout life. Its ligands are expressed in surrounding mesenchyme and can, in the absence of exogenous mechanical cues, induce progenitors to form alveolospheres with intermingled AT2 and AT1 cells. FGF signaling directly and cell autonomously specifies AT2 fate; progenitors lacking *Fgfr2* in vitro and in vivo exclusively acquire AT1 fate. *Fgfr2* loss in AT2 cells perinatally results in reprogramming to AT1 identity, whereas loss or inhibition later in life triggers AT2 apoptosis and compensatory regeneration. We propose that Fgfr2 signaling selects AT2 fate during development, induces a cell non-autonomous AT1 differentiation signal, then continuously maintains AT2 identity and survival throughout life.

## Abstract (web summary)

Here the authors show that FGF signaling initiates alveolus development in mouse lung by inducing AT2 fate and a secondary signal for AT1 fate, and continuously maintains AT2 cells throughout life. FGF inhibition triggers immediate AT2 apoptosis and compensatory regeneration.

## Introduction

Gas exchange occurs in alveoli, tiny terminal air sacs of the lung lined by two intermingled epithelial cell types: exquisitely thin alveolar type 1 (AT1) cells that provide the gas-exchange surface, and cuboidal AT2 cells that secrete surfactant to prevent alveolar collapse^1^. Alveoli are also the site of some of the most significant but poorly understood and difficult-to-treat human diseases. These include bronchopulmonary dysplasia in infants, COPD/emphysema^2^, pulmonary fibrosis^3^, adenocarcinoma^4,5^, and infections including severe acute respiratory syndrome (SARS)^6,7^ and COVID-19^8^ in adults. Understanding how these key alveolar cell types arise during development and are maintained throughout life is critical for understanding and treating these diseases, and for guiding developmental, regenerative and tissue engineering approaches to create healthy alveoli and the surfactant that lines them.

Several lines of evidence, including marker expression, lineage tracing, and clonal analysis in mice, indicate that AT1 and AT2 cells arise directly from a common progenitor during embryonic development^5,9^. This model is supported by single-cell RNA sequencing studies of developmental intermediates that reconstructed the full, bifurcating gene expression program from bipotent progenitors at embryonic day 16.5 (e16.5) to either AT1 or AT2 cells over the next several days of fetal development^10–12^, although a recent genetic labeling study claims an earlier fate commitment^13^. Multiple developmental signaling pathways^14–18^ and transcription factors^19^ can influence alveolar structure, maturation, or the balance of the two cell types^20–22^, but the key driver of alveolar fate selection and differentiation is thought to be mechanical forces^23^. The build-up and increased movement of luminal fluid in late gestation is proposed to stretch and flatten progenitors into AT1 cells^18,24,25^, as stretch does to cultured AT2 cells to cause AT1 transdifferentiation in vitro^26^. Live imaging indicates that alveolar progenitors protected from luminal mechanical forces during budding default to the AT2 cell program^18^.

Here we demonstrate that alveolar cell fate specification is, in fact, dictated by a classic growth factor signal, the same FGF signaling pathway that controls the earlier steps of airway budding and branching^22,27–30^ and also initiates subsequent budding^18^. After branching, the receptor Fgfr2 remains on in alveolar progenitors and dynamically restricts to the AT2 lineage shortly before cellular differentiation, while its ligands Fgf7 and Fgf10 continue to be expressed by surrounding stromal cells. We show by the effects of the ligands on isolated e16.5 progenitors in cultures lacking budding and differential mechanical cues, and by genetic mosaic and pharmacological studies in culture and in vivo, that Fgfr2 signaling directly and cell autonomously specifies AT2 fate while inducing a secondary, non-autonomous signal that promotes AT1 fate of neighboring progenitors. This developmental FGF pathway remains on throughout life, where it serves continuously and ubiquitously to maintain healthy AT2 cells^31,32^, which, if deprived of the FGF signal early in life, reprogram to AT1 fate and later in life, immediately undergo apoptosis.

## Results

### Fgfr2 restricts to the AT2 lineage during alveolar differentiation

**Fig. 1:**
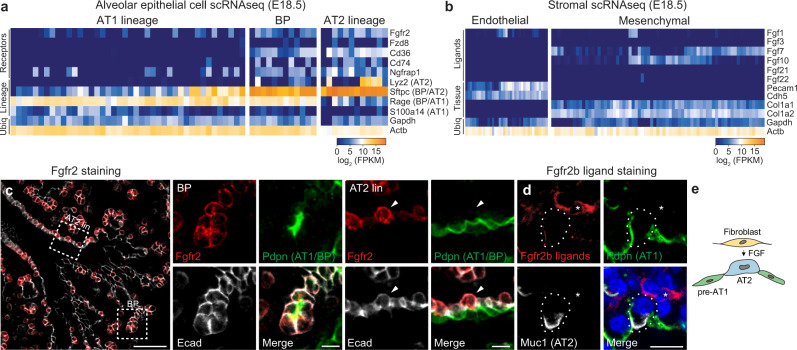
Expression of Fgfr2 and its ligands during alveolar differentiation. **a** Expression of *Fgfr2* and the four other receptor genes most selectively expressed in alveolar type 2 (AT2) cell lineage (“Receptors”) from single-cell RNA sequencing (scRNAseq) analysis of distal (alveolar) epithelial cells from embryonic day 18.5 (e18.5) mouse lung. Cells (columns) are arranged in developmental pseudotime (BP, bipotent progenitors, center; AT1 lineage to the left; AT2 lineage to the right) determined as described in ref. 11 by expression of alveolar lineage markers like the four shown (“Lineage”) including two that restrict from BP to AT2 lineage (BP/AT2, *Sftpc*) or to AT1 lineage (BP/AT1, *Rage*). (Note due to the very high expression of *Sftpc* in bipotent progenitors, there is a temporal lag in the extinction of the transcripts in newly-differentiating AT1 cells and thus some nascent AT1 cells co-express *Sftpc* and *Rage*.) Ubiq, ubiquitously-expressed control genes. Heat map, mRNA expression level. **b** Expression of Fgfr2b ligand genes in distal (alveolar) endothelium and mesenchyme from scRNAseq of e18.5 lung. **c** e17.5 lung immunostained for Fgfr2 (red), bipotent progenitor (BP) and AT1 marker podoplanin (Pdpn, green), and epithelial marker E-cadherin (E-cad, white). Boxed regions, close-ups, and split channels at the right of the bipotent progenitor (BP) and developing AT2 lineage cell (AT2 lin). Note in BP close-up BPs are cuboidal, Pdpn-expressing cells. In AT2 lin closeup, Fgfr2 (red) remains on in developing AT2 cells (arrowhead) but downregulated in neighboring developing AT1 cells, and although BP/AT1 marker (Pdpn) is still detected it too will soon be downregulated as developing AT2 cell matures (see Supplementary Fig. 1a). Bars, 50 µm (left panel), 10 µm (close-ups). Biological triplicates were analyzed. **d** e17.5 lung stained with the Fgfr2 (isoform iiib) ligand-binding domain fused to human IgG_1_ domain to show Fgfr2b ligands (red), and co-stained for Pdpn (green), and AT2 marker mucin1 (Muc1, white). Fgfr2b ligands are detected in nearby mesenchymal cells (asterisk) and diffusely around developing alveolar epithelial cells. Bar, 10 µm. Repeated in biological triplicate. **e** Schematic showing inferred signaling from Fgf ligand-expressing mesenchymal cell (fibroblast) to nearby Fgfr2-expressing nascent AT2 cell during alveologenesis. pre-AT1, nascent AT1 cells that downregulated Fgfr2. All experiments were repeated at least three times.

To identify signaling pathways that might control alveolar cell fate selection, we searched the single-cell transcriptional program of mouse alveolar development^11^ for receptor genes enriched in either the AT1 or AT2 lineages. Of the five receptor genes showing at least twofold enrichment in the AT2 lineage (Fig. 1a; *Fgfr2*, *Fzd8*, *Cd36*, *Cd74*, and *Ngfrap1*), only *Fgfr2* is known to be required for embryonic lung development^22,30^. The other four either have no known consequence (*Fzd8*), only adult lung phenotypes (*Cd36* and *Cd74*), or have not yet been examined (*Ngfrap1*). *Fgfr2* is expressed early and throughout the developing lung epithelium and its deletion abrogates airway branching^22,29,30^, so its later function has been more challenging to investigate^14,31–33^. The single-cell RNA sequencing (scRNAseq) profiles demonstrated that the iiib isoform of *Fgfr2* is expressed in bipotent progenitors and maintained in the AT2 lineage but downregulated in the AT1 lineage (Fig. 1a and Supplementary Fig. 1c). Fgfr2 immunostaining during mouse alveolar development confirmed that Fgfr2 is expressed in bipotent progenitors (Fig. 1c), downregulated in nascent AT1 cells, and maintained in nascent AT2 cells as they bud into the surrounding mesenchyme and become histologically and molecularly distinct from AT1 cells (Fig. 1c and Supplementary Fig. 1a). Indeed, Fgfr2 is among the earliest known markers of AT2 fate selection.

To identify the relevant Fgfr2 ligands, we performed scRNAseq on the adjacent mesenchymal cells during alveolar differentiation. Two Fgfr2 (isoform iiib) ligands, *Fgf7* and *Fgf10*, were detected in mesenchymal cells (Fig. 1b), predominantly in the previously identified major subpopulation (“matrix fibroblast”) marked by *Wnt2* expression (Supplementary Fig. 2a)^10^. Both *Fgf* genes are also expressed in lung mesenchyme earlier in development, when Fgf10 serves as the key ligand in Fgfr2-induced branching of the bronchial tree^22,27^. Visualization of Fgfr2 ligands with the Fgfr2 (isoform iiib) ligand-binding domain fused to the human IgG_1_ domain showed ligand production in a subset of mesenchymal cells near budding AT2 cells (Fig. 1d, e and Supplementary Fig. 1b).

### Fgfr2 ligands induce alveolar differentiation in culture

**Fig. 2:**
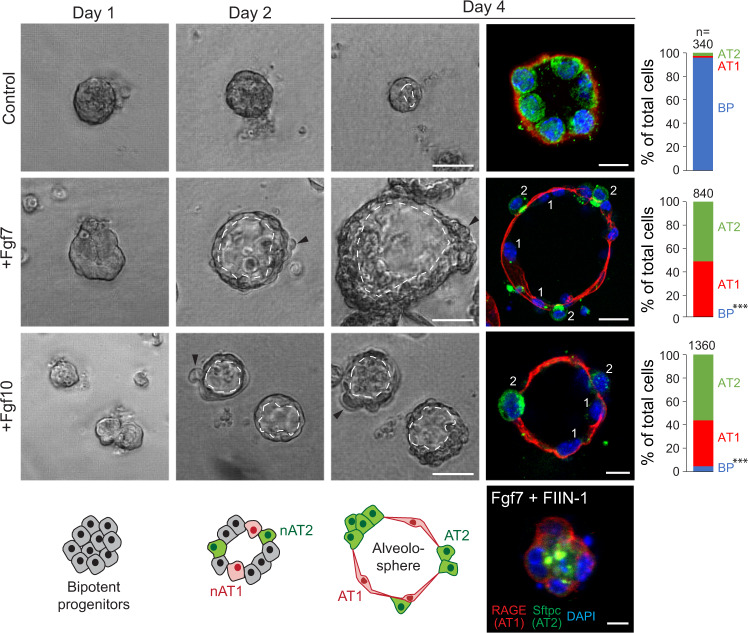
Effect of Fgfr2 ligands on purified alveolar epithelial progenitors in culture. Phase images (left panels) and immunostains (right) of epithelial progenitors purified from tips of e16.5 lungs and cultured in Matrigel for the period indicated with media alone (Control, top panels) or media supplemented every two days with Fgf7 (50 ng/ml, middle panels) or Fgf10 (100 ng/ml) and HSPG (100 ng/ml, lower panels). Note an increase in luminal surface area (dashed lines) and basal extrusion (arrowheads) in cultures with Fgf ligands, and differentiation into intermingled cuboidal AT2 (Sftpc^pos^, green) and squamous AT1 cells (RAGE^pos^, red) at day 4 (“alveolospheres”). In control cultures lacking FGFs (top row) or treated both with Fgf7 and the Fgfr inhibitor FIIN-1 (bottom panel), cells remained bipotent progenitors throughout, as shown by co-expression of both markers. nAT1, nascent AT1 cell; nAT2, nascent AT2 cell. Bars, 50 µm (left panels and right +Fgf7 panel), 10 µm (right top and +Fgf10 panels), and 10 µm (Fgf7 + FIIN-1 panel). Co-treatment with both Fgf7 and Fgf10 (with HSPG) gave similar results as each treatment alone. Quantification at right gives the percent of each cell type (BP), AT1, AT2) in the culture at day 4 determined by immunostaining (*n* = 348 cells scored for control, 843 for Fgf7-treatment, and 1365 for Fgf10-treatment in experimental triplicate). ***p = 7.7 × 10^−28^ for Fgf7-treatment and 6.4 × 10^−26^ for Fgf10-treatment (Student’s two-sided *t*-test), BP abundance of either Fgf treatment condition vs control.

To explore the function of Fgfr2 signaling in alveolar development, we first examined the effect of Fgf7 and Fgf10 on purified epithelial progenitors isolated from the tips of e16.5 lungs, before AT1 and AT2 cells are detected. When the progenitors were cultured for up to 8 days in Matrigel in the absence of exogenous FGFs, they failed to develop into either AT1 or AT2 cells, remaining cuboidal and organizing into multicellular clusters around a lumen (Fig. 2 and Supplementary Fig. 3c). By contrast, in the presence of Fgf7 (50 ng/ml), the progenitors organized into large epithelial spheres containing differentiated cuboidal Sftpc^pos^ Rage^neg^ AT2 cells intermingled with squamous Sftpc^neg^ Rage^pos^ Pdpn^pos^ AT1 cells, similar to the structures formed during alveologenesis in vivo beginning at ~e17.5 (Fig. 2 and Supplementary Fig. 3b). Fgf10 induced formation of similar epithelial spheres composed of intermingled AT2 and AT1 cells when administered with heparan sulfate proteoglycans (HSPGs), a co-receptor that increases ligand accessibility^34,35^ (Fig. 2). The effects of both Fgf7 and Fgf10 were abrogated in the presence of 10 nM Fgfr inhibitor FIIN-1 (Fig. 2 and Supplementary Fig. 3a).

Live imaging of the cultures showed that Fgf7 stimulated continuous growth of the epithelial spheres (Supplementary Fig. 3f); however, no early cell budding like that proposed to protect progenitors from luminal mechanical forces during alveolar development in vivo^18^ was detected. Transient buds were occasionally observed, but only after luminal expansion and AT1 flattening were apparent (Supplementary Fig. 3g). The Arp2/3 inhibitor CK666, used to block protrusions and AT2 development in vivo^18^, reduced the transient buds but not Fgf7-induced AT1 and AT2 development in culture (Supplementary Fig. 3h). Thus, Fgfr2 signaling drives differentiation of alveolar progenitors and formation and growth of alveolus-like structures (“alveolospheres”) with intermingled AT1 and AT2 cells, and it does so independent of extrinsic mechanical forces and without the early budding and associated structural features proposed to differentially protect progenitors from such forces. However, it is possible that extrinsic mechanical forces and budding in vivo might regulate some aspect of Fgf signaling or the developmental process that is dispensable in the culture system.

### Fgfr2 signaling directly selects AT2 cell fate

**Fig. 3:**
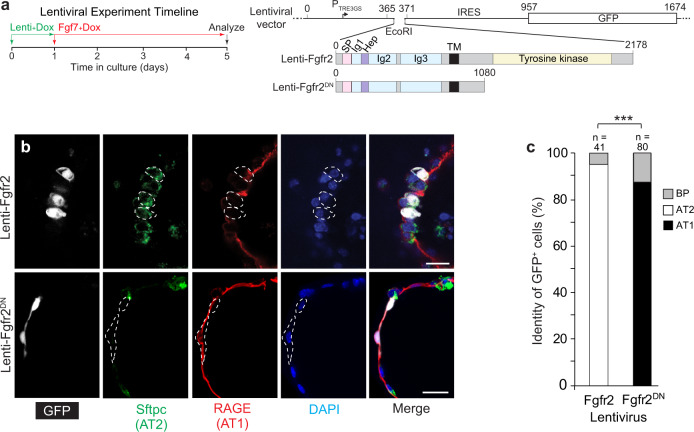
Fgfr2 signaling controls AT2 fate selection cell autonomously in culture. **a** Experiment timeline and structures of lentiviral vectors co-expressing wild-type Fgfr2 (Lenti-Fgfr2) or dominant negative Fgfr2 lacking the tyrosine kinase domain (Lenti-Fgfr2^DN^) and GFP. Numbers above the vector indicate nucleotide position and above Fgfr2 structure indicate amino acid residue; colors highlight functional domains in Fgfr2. TRE tetracycline responsive element, IRES internal ribosome entry site, GFP green fluorescent protein, SP signal peptide, Ig immunoglobulin domain, Hep heparin sulfate binding domain, TM transmembrane domain, DN dominant negative. **b** e16.5 alveolar progenitors were mosaically infected (less than 1% of the cultured cells were infected) with the lentiviral vectors indicated at the time of cell plating in doxycycline-containing media (100 ng/ml) for 24 h, then cultured with Fgf7 and doxycycline as in Fig. 2 for 4 days and immunostained for markers indicated. Note that GFP^pos^ cells in top panel with Fgfr2 signaling promoted cell autonomously by expression of wild-type Fgfr2 became Sftpc^pos^ RAGE^neg^ cuboidal AT2 cells, whereas GFP^pos^ cells in the bottom panel with Fgfr2 signaling cell autonomously inhibited by expression of Fgfr2^DN^ became Sftpc^neg^ RAGE^pos^ squamous AT1 cells. See Supplementary Fig. 4A for images of rare infected cells that did not acquire the predominant fate. Bars, 20 µm. **c** Quantification of **b**. Sftpc^pos^ RAGE^pos^ cuboidal cells were scored as bipotent progenitors (BP). *n* number of GFP^pos^ cells scored in three experiments. ***p = 4.0 × 10^−13^ for Lenti-Fgfr2 and 2.2 × 10^−16^ for Lenti-Fgfr2^DN^ (chi-squared). All experiments were repeated at least three times.

The addition of FGF ligands to the cultures triggered the differentiation of progenitors into both AT1 and AT2 cells. To distinguish direct (cell autonomous) effects of FGF from secondary (cell non-autonomous) effects, we devised a strategy to enforce or block Fgfr2 signaling in random subsets of cultured progenitors targeted by a lentiviral vector. The vectors expressed either wild-type Fgfr2 (Lenti-Fgfr2) or a dominant negative form lacking the cytoplasmic domain (Lenti-Fgfr2^DN^) along with a fluorescent reporter (GFP) to mark infected cells (Fig. 3a). We found that nearly every progenitor cell (95%) infected with virus expressing wild-type Fgfr2 differentiated into an AT2 cell, and conversely infected cells expressing Fgfr2^DN^ almost always (88%) acquired AT1 fate (Fig. 3b, c); the rare cells that did not acquire the predominant fate remained undifferentiated (Fig. 3c and Supplementary Fig. 4a). Experiments with a control virus (AAV-nGFP) that expresses GFP but no Fgfr showed that randomly-infected progenitors have a roughly equal chance of acquiring AT1 or AT2 fate during culturing (Supplementary Fig. 4b). These results, together with the above result showing e16.5 distal progenitors cultured for over a week in the absence of FGF fail to differentiate into AT1 or AT2 cells (Fig. 2 and Supplementary Fig. 3c), indicate that the fate of alveolar progenitors is not committed at e16.5, countering claims of an earlier fate commitment^13^ but consistent with prior lineage tracing^9^, clonal analysis^5^, and scRNAseq results^10–12^. We conclude that distal epithelial progenitors at e16.5 are indeed bipotent, having the capacity to differentiate into either AT1 or AT2 cells, and that AT2 fate is selected directly and cell autonomously by Fgfr2 signaling. The results also imply that the observed induction of AT1 fate by FGF addition to the cultures must be indirect (cell non-autonomous), presumably via a secondary signal produced by maturing AT2 cells and received by neighboring progenitors (see Discussion). AT1 differentiation cannot simply be the default fate because it, too, requires the addition of FGF ligands to the culture.

**Fig. 4:**
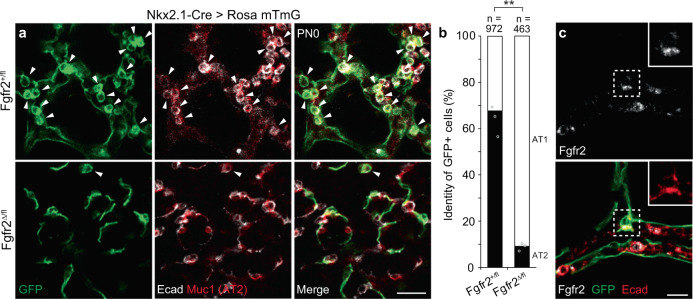
Cell-autonomous requirement of *Fgfr2* for AT2 fate selection in vivo. **a** Alveolar region of lungs from *Nkx2.1-Cre; Rosa26-mTmG; Fgfr2*^*fl/+*^ (upper panels) or *Nkx2.1-Cre; Rosa26-mTmG; Fgfr2*^*fl/delta*^ (lower) mice at postnatal day 0 with Cre expressed in developing lung epithelial cells to delete conditional *Fgfr2* allele (*Fgfr2*^*fl*^) and activate a farnesylated GFP protein reporter, which targets all membranes including cytoplasmic vesicles (mTmG, green). Lungs were immunostained for GFP, E-cadherin (E-cad), and AT2 marker Muc1 as indicated. Note mosaic GFP expression showing Cre is active in some but not all alveolar epithelial cells, and that *Fgfr2* is required cell autonomously for AT2 cell development because GFP^pos^ control cells carrying wild-type *Fgfr2* allele (*Fgfr2*^*fl/+*^, upper panels) became either cuboidal Muc1^pos^ AT2 cells (arrowheads) or squamous AT1 cells, whereas GFP^pos^ cells lacking *Fgfr2* (*Fgfr2*^*fl/delta*^, lower panels) almost exclusively became AT1 cells. Bar, 50 µm. **b** Quantification of **a**. *n* number of GFP^pos^ cells scored in three lungs. **p = 0.0022 (Student’s two-sided *t*-test, data as mean ± SD). **c** Close-up of a rare GFP^pos^ AT2 cell (boxed) from *Nkx2.1-Cre; Rosa26-mTmG; Fgfr2*^*fl/delta*^ lung, as in bottom panels of **a**, stained for Fgfr2 and markers indicated. Note that Fgfr2 has not yet been lost from the GFP^pos^ cell, implying the recent deletion of conditional allele *Fgfr2*^*fl*^ and perdurance of Fgfr2 that promoted AT2 fate selection. Bar, 10 µm. Stain repeated in biological triplicate. All experiments were repeated at least three times.

To determine if Fgfr2 signaling plays a similar role in alveolar cell fate selection in vivo, we used an *Nkx2.1-Cre* transgene with low Cre activity to mosaically delete *Fgfr2* in the developing lung epithelium, combined with a fluorescent Cre reporter allele (*Rosa26*^*mTmG*^) to mark the cells in which Cre was active (Fig. 4 and Supplementary Fig. 4c). In control mice carrying a wild-type *Fgfr2* allele in trans to the conditional *Fgfr2* allele (*Tg*^*Nkx2.1-Cre*^*;Fgfr2*^*fl/+*^*; Rosa26*^*mTmG/+*^), one-third of distal cells with Cre reporter activity (36%, *n* = 970 GFP + cells scored in three mice) acquired AT1 fate by the end of fetal life (PN0) and the rest acquired AT2 fate (64%) (Fig. 4a, b). By contrast, in animals with an *Fgfr2* deletion in trans to the conditional allele (*Tg*^*Nkx2.1Cre*^*;Fgfr2*^*fl/del*^*;Rosa26*^*mTmG/+*^), the targeted distal cells almost exclusively (91%, *n* = 460 GFP + cells scored in three mice) acquired AT1 fate (Fig. 4a, b); the few cells that did not acquire AT1 fate had not yet lost Fgfr2 expression (Fig. 4c). We conclude that *Fgfr2* is the critical determinant of AT2 fate and is required cell autonomously for AT2 fate selection in vivo as well as in vitro, and that alveolar progenitors lacking Fgfr2 signaling acquire the alternative, AT1 fate.

### Fgfr2 signaling prevents AT2 reprogramming to AT1 fate in juvenile life

**Fig. 5:**
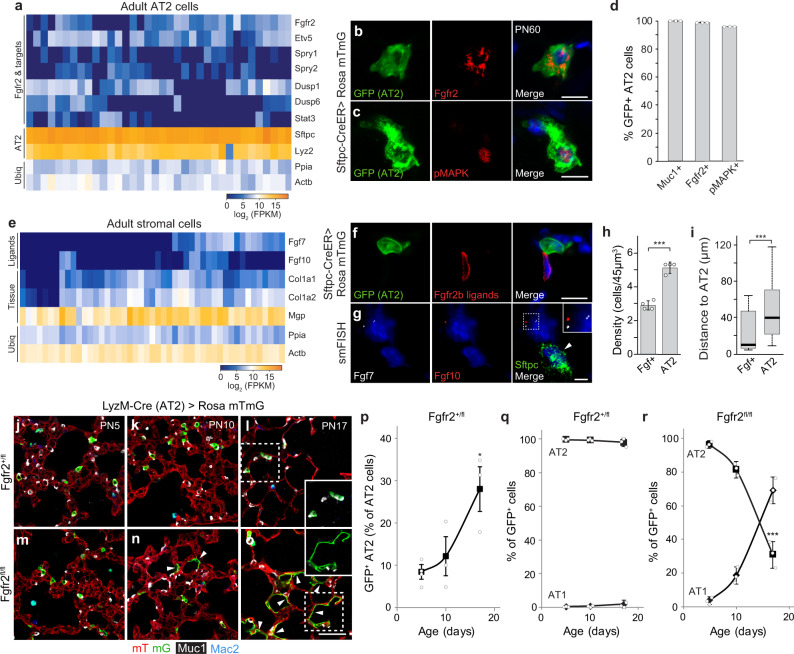
Fgfr2 pathway prevents AT2 reprogramming to AT1 after birth. **a** scRNAseq of adult AT2 cells. Expression of *Fgfr2*, downstream transcription factor *Etv5*, and target genes indicates pathway remains on after birth. AT2, AT2 markers; Ubiq, ubiquitously-expressed genes. **b**, **c** AT2 in adult (PN60) *Sftpc-CreER;Rosa26-mTmG* mouse tamoxifen-induced to label AT2 (GFP) and co-stained for Fgfr2 (**b**) or phosphorylated MAP kinase (pMAPK) to show pathway activity (**c**). Blue, DAPI counterstain. Bars, 10 µm. **d** Quantification of **b**, **c**. Nearly all labeled cells (GFP^+^) express AT2 marker (Muc1) and Fgfr2, and show Fgfr2 activity (pMAPK^+^). *n* = 300 GFP^+^ cells scored (three animals). **e** Fgfr2 ligand expression (scRNAseq) in lung stromal cells. **f** AT2 in adult *Sftpc-CreER;Rosa26-mTmG* mouse tamoxifen-induced to label AT2 (GFP^+^) and co-stained for Fgfr2 (isoform iiib) ligand-binding domain to show Fgfr2 ligands (ligands identified in Supplementary Fig. 2). Blue, DAPI. Bar, 10 µm. **g** smFISH (*Fgf7*, *Fgf10*, *Sftpc*) in adult (PN60) lung. Cell neighboring AT2 co-expresses *Fgf7* (white dots) and *Fgf10* (red dots) (enlarged in inset). Blue, DAPI; arrowhead, AT2. Bar, 5 µm. **h**, **i** Quantification of **g** showing the density of *Fgf*-expressing and AT2 cells (**h**, ***p = 4.8 × 10^−5^, Student’s two-sided *t*-test, data shown as mean ± SD) and distance between them (**i**, ***p = 2.9 × 10^−50^, Mann Whitney, boxplot shows minimum, maximum, first quartile, second quartile (median), and third quartile). *n* = 182 AT2, 103 Fgf^+^ cells scored (four animals). (Values indicate Fgf availability and how many AT2 it supports, and how far each Fgf-expressing cell must extend (or ligand diffuse) for support). **j**–**o** Alveolar region of *LyzM-Cre;Rosa26-mTmG;Fgfr2*^*fl/+*^ (**j**–**l**) or *LyzM-Cre;Rosa26-mTmG;Fgfr2*^*fl/fl*^ (**m**–**o**) mice (ages indicated) immunostained for Cre reporter (mTmG), AT2 (Muc1), and macrophage (Mac2, distinguishes GFP-labeled macrophages) markers. *LyzM-Cre* becomes active in AT2 in early postnatal life, activating the GFP reporter and deleting *Fgfr2*^*fl*^. Control GFP^pos^ AT2 cells carry a wild-type *Fgfr2* allele (*Fgfr2*^*fl/+*^, **j**–**l**) and remain AT2 (inset in **l**), whereas those lacking *Fgfr2* (*Fgfr2*^*fl/fl*^, **m**–**o**) convert to AT1 (arrowheads, inset in **o**). Bar, 50 µm. **p**–**r** Quantification (mean ± SD) of **j**–**o** showing increasing Cre activity in AT2 in early postnatal life (**p**) and identities of GFP^pos^ cells in control (**q**) and following *Fgfr2* loss (**r**). *n* = 300 GFP^pos^ cells scored (three animals) per timepoint/condition; *p = 0.01 for (**p**) and ***p = 0.0002 for (**r**) (Student’s two-sided *t*-test, time series end vs. start).

Curiously, this developmental signaling pathway remains on later in life, for months or even years, after alveoli have acquired their canonical structure and function. *Fgfr2* continues to be selectively expressed in AT2 cells (Fig. 5a, b, d and Supplementary Fig. 5), *Fgf7* and *Fgf10* continue to be expressed in *Wnt2*-expressing alveolar fibroblasts and at lower levels in myofibroblasts (Fig. 5e–i and Supplementary Fig. 2b, c), and the Fgfr2 signaling pathway remains active in mature AT2 cells as detected by phosphorylated MAP kinase (Fig. 5c, d) and expression of Fgfr2-induced genes such as *Spry2* (Fig. 5a). To investigate the role of this selective and persistent Fgfr2 signaling in AT2 cells, we first used a *Cre* knock-in allele at the endogenous *LysozymeM* (*LyzM*, also known as *Lyz2*) locus, which turns on in some AT2 cells early in postnatal life and is progressively activated later in other AT2 cells^5^ (see below), to conditionally delete *Fgfr2* in AT2 cells at different ages. There was a dramatic effect of *Fgfr2* deletion from AT2 cells during juvenile life and an equally significant but entirely different effect in adults.

**Fig. 6:**
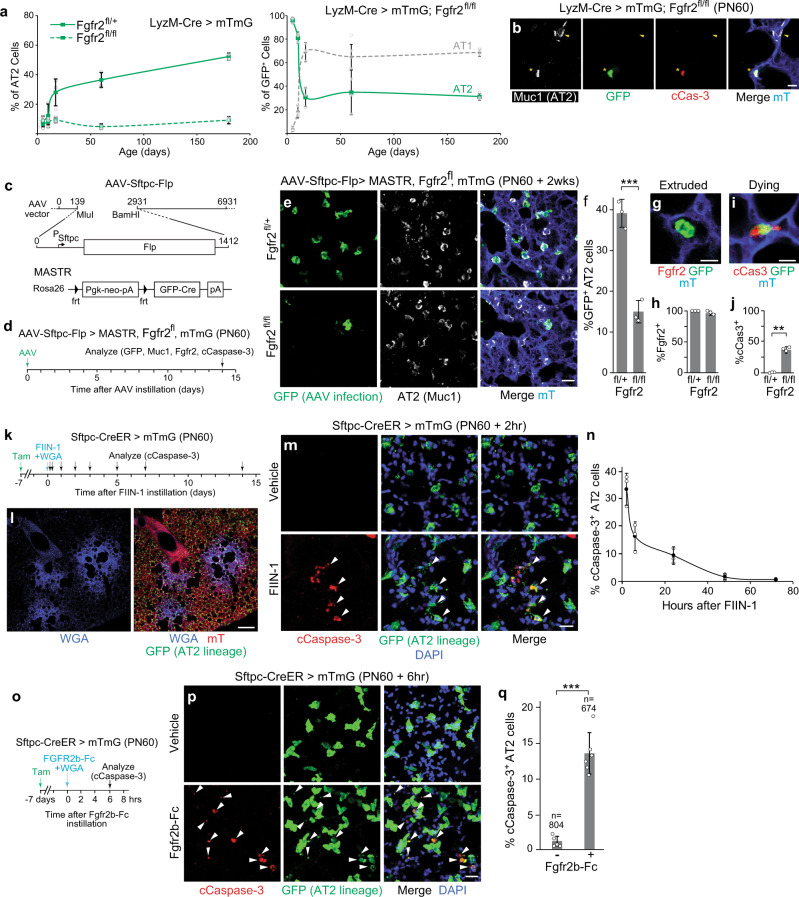
*Fgfr2* signaling continuously prevents adult AT2 apoptosis. **a** (Left) Time course showing the proportion of AT2 (Muc1^+^) cells labeled with GFP in control (*LyzM-Cre; Rosa26-mTmG Fgfr2*^*fl/+*^) or *Fgfr2* deleted (*LyzM-Cre; Rosa26-mTmG; Fgfr2*^*fl/fl*^) lungs in postnatal life (control, extension of Fig. 5p, mean values ± SD). No further accumulation of GFP^pos^ AT2 occurs in *Fgfr2* deleted lungs (dashed line) after PN20, despite continued LyzM-Cre activity (AT2 labeling) apparent in control (solid line). *n* = 3955 AT2 (3 animals/genotype). (Right) Time course showing identities of GFP^pos^ cells following *Fgfr2* loss (extension of Fig. 5r). After a rapid increase in AT1 due to AT2 reprogramming in the juvenile period (through PN20), no further accumulation of GFP^pos^ AT1 or AT2 occurs. *n* = 300 GFP^pos^ cells scored (3 animals/condition and timepoint, mean ± SD). **b** Close-up of the alveolar region in adult (PN60) *LyzM-Cre;Rosa26-mTmG;Fgfr2*^*fl/fl*^ mouse stained for apoptosis marker cleaved Caspase-3 (cCaspase-3). Note GFP^pos^ AT2 deleted for *Fgfr2* undergoing apoptosis (asterisk) but not unlabeled control AT2 nearby (arrowhead). Bar, 10 µm. **c**–**f** MASTR system for the conditional, complete deletion of *Fgfr2* in AT2. **c** AAV-Sftpc-Flp virus (top) expresses Flp from *Sftpc* promoter. MASTR allele (bottom), following Flp-mediated deletion (at frt, triangles) of *Pgk-neo-pA* cassette, constitutively expresses GFP-Cre fusion. **d** Scheme. Two weeks after AAV-Sftpc-Flp instillation into lungs of adult *Fgfr2*^*fl/fl*^*;Rosa26*^*mTmG/MASTR*^ mice to induce constitutive GFP-Cre and complete deletion of *Fgfr2*^*fl/fl*^ in AT2, lungs were immunostained (GFP, Muc1, Fgfr2, cCaspase-3). **e** Alveolar regions from *Fgfr2*^*fl/+*^ control (top) or *Fgfr2*^fl/fl^ (bottom) mice treated as in (**d**). Note the reduction in GFP^pos^ AT2 (Muc1^pos^) (bottom). Bar, 25 µm. **f** Quantification of AT2 loss in (**e**). *n* = 890 Muc1^pos^ cells scored/condition (three experiments, mean ± SD). **g**, **h** Quantification (**h**) of Fgfr2 immunostaining (*n* = 100 GFP^pos^ cells scored/condition, three experiments, mean ± SD). Nearly all (96%) GFP^pos^ AT2 remaining after MASTR-mediated *Fgfr2*^*fl/fl*^ deletion (**e**, **f**) have not fully lost Fgfr2. Micrograph (**g**) shows an example of rare GFP^pos^Fgfr2^neg^ AT2, which were all rounded and extruded into the lumen. Bar, 10 µm. **i**, **j** Quantification (**j**) of cCaspase-3 immunostaining (*n* = 470 GFP^pos^ cells scored/condition, mean ± SD). Note that 36% of GFP^pos^ AT2 cells remaining after MASTR-mediated *Fgfr2*^*fl/fl*^ deletion were cCaspase-3^pos^, indicating apoptosis initiated (micrograph, **i**). Bar, 10 µm. **k**–**n** Acute inhibition of Fgfr2 signaling by FIIN-1. **k** Scheme for co-instillation of FIIN-1 and fluorescent marker WGA-405 into lungs of adult *Sftpc*^*CreER/+*^*;Rosa26*^*mTmG/+*^ mice tamoxifen(Tam)-treated to label AT2 then analyzed over 2 weeks. Bar, 200 µm. **l** Lung regions exposed to FIIN-1 (WGA-labeled, left) and shown merged (right) with mTmG fluorescence (AT2 lineage GFP-labeled, other cells tdTomato (mT)-labeled). Bar, 250 µm. **m** WGA-labeled alveolar regions exposed 2 h to the vehicle (top) or FIIN-1 (bottom). FIIN-1-exposed AT2 cells (GFP^pos^) undergo apoptosis (cCaspase-3^pos^, arrowheads). Bar, 20 µm. **n** Kinetics of AT2 apoptosis in (**m**). In vehicle control, <2.5% of AT2 were cCaspase-3^pos^ (*n* = 650 cells scored/condition, three experiments, mean ± SD). No conversion of labeled AT2 to AT1 was detected during this period (*n* = 390 cells scored/condition, three experiments) (see also Suppl. Fig. 7). **o**–**q** Effect of acute Fgfr2 inhibition in AT2 cells by Fgfr2iiib-Fc protein. **o** Scheme. After Tam-induction of adult *Sftpc*^*CreER/+*^*;Rosa26*^*mTmG/+*^ mice to GFP-label AT2, Fgfr2iiib-Fc (or vehicle) and WGA-405 were co-instilled and lungs subsequently stained for cCaspase-3. **p** WGA-labeled alveolar regions from vehicle control (top) and Fgfr2iiib-Fc (bottom) instilled lungs. Bar, 25 µm. **q** Quantification of **p**. *n*, cells scored, six experiments (mean ± SD). ***p = 4.3 × 10^−4^ for (**f**), **p = 3.4 × 10^−3^ for (**j**), and ***p = 2.5 × 10^−4^ for (**q**) (Student’s two-sided *t*-test) (panels **f**, **j**, **q**).

LyzM-Cre activity is first detectable in rare AT2 cells at PN5, as shown by the Cre reporter (Fig. 5j), and then gradually becomes active in additional AT2 cells (Figs. 5p, 6a (left panel). In juvenile control mice carrying an *Fgfr2* wild-type allele (*LyzM*^*Cre/+*^*;Fgfr2*^*fl/+*^*;Rosa26*^*mTmG/+*^), nearly all (>96%) AT2 cells expressing the Cre reporter retained their AT2 identity (Fig. 5j–l, q), as previously observed for *Fgfr2*^*+/+*^ AT2 cells^5^. By contrast, in juvenile mice homozygous for the *Fgfr2* conditional allele (*LyzM*^*cre/+*^*Fgfr2*^*fl/fl*^*;Rosa26*^*mTmG/+*^), there was a rapid decline in the proportion of reporter-expressing cells with AT2 identity, from 96% at PN5 to 31% at PN17, and a concurrent increase in labeled cells that had acquired AT1 fate, from 3.8% at PN5 to 69% at PN17 (Fig. 5m–o, r). *Fgfr2* deletion in AT2 at PN0 using *Sftpc-CreER* can also promote AT1 fate^36^. A similar result was obtained when Fgfr2 signaling was inhibited by the addition of FIIN-1 to newly formed AT1 and AT2 cells generated from bipotent progenitors cells in culture, resulting in an increase in AT1 and reduction in AT2 cells (Supplementary Fig. 3d, e). We conclude that Fgfr2 signaling is necessary to maintain AT2 fate in newly formed AT2 cells during juvenile life, and that loss of Fgfr2 signaling during this period results in rapid and robust reprogramming to AT1 fate.

### *Fgfr2* signaling continuously prevents AT2 apoptosis during adult life

After the juvenile period (PN17) in the above experiment, there was no further increase in AT2 lineage-labeled cells with AT1 fate (Fig. 6a, right panel and Supplementary Fig. 6), indicating that AT2 cells that lose *Fgfr2* in adult life do not reprogram to AT1 fate. There was also no further accumulation of lineage-labeled AT2 cells during this period (Fig. 6a, right panel), suggesting that *Fgfr2* deletion in adult AT2 cells results in their loss. Indeed, we occasionally detected expression of cleaved Caspase-3 in such cells, indicating they had initiated apoptosis (Fig. 6b). Recently, others have noted a variable reduction in AT2 cell markers or abundance and impaired recovery from severe lung injury when one or more *Fgfr* genes were conditionally deleted^31–33^, attributing the deficits to a reduction in stem cells^31^ or their proliferation^32,33^. However, another recent study reported that *Fgfr2* in AT2 cells is entirely dispensable during homeostasis^36^. To more precisely determine the cellular and molecular consequences of acute abrogation of FGF signaling in adult AT2 cells, we first tried a tamoxifen-inducible Cre recombinase *(Sftpc*^*CreER/+*^*; Fgfr2*^*del/fl*^*; Rosa26*^*mTmG/+*^) but found that removal of *Fgfr2* occurred but was inefficient under these conditions (Supplementary Fig. 5c). We, therefore, developed three additional approaches, which revealed that Fgfr2 signaling is ubiquitously and continuously required for AT2 cell maintenance throughout adult life, and even brief deprivation of signaling immediately initiates apoptosis.

Two important limitations of the conditional *Fgfr2* deletion experiment noted above were that the gene was inefficiently deleted, and even when the gene was deleted, the protein persisted (perdured), limitations that could have contributed to the modest or entire lack of effect observed in other studies^32,36^. To overcome the former limitation, we designed a combinatorial genetic approach using the MASTR transgene^37^ (Fig. 6c), which following Flp-mediated recombination of the transgene provides constitutive and high-level Cre expression to ensure recombination of all Cre-dependent alleles in the cell, such as *Fgfr2*^*fl*^ and *Rosa26*^*mTmG*^. We used the MASTR allele in conjunction with an AAV virus we engineered to express Flp recombinase specifically in AT2 cells using an *Sftpc* promoter element (Fig. 6c, d); when the virus was instilled intratracheally into adult control mice (*Fgfr2*^*+/fl*^*; Rosa26*^*mTmG/MASTR*^), specific expression of Cre-dependent reporter genes was observed in AT2 cells (Fig. 6e; >98% of GFP^+^ cells, *n* = 3 lungs, 168 GFP^+^ cells counted per lung at 2 weeks), similar to the AT2 specificity observed in prior studies with this approach^38–40^. When the virus was instead instilled into *Fgfr2*^*fl/fl*^*; Rosa26*^*mTmG/MASTR*^ mice, 60% fewer GFP^+^ AT2 cells were observed (Fig. 6e, f); of the GFP^+^ AT2 cells that remained, nearly all (96%) were found to have retained Fgfr2 protein due to perdurance (Fig. 6h), and the rare GFP^+^ Fgfr2^-^ AT2 cells detected (4 of 96 scored GFP^+^ AT2 cells from three mice) had been extruded into the alveolar space and had a rounded morphology (Fig. 6g). This result indicates that adult AT2 cells broadly and uniformly undergo apoptosis following removal of Fgfr2. Apoptosis may even initiate with only partial reduction in Fgfr2 levels because one-third (36%) of the remaining GFP^+^ AT2 cells in this experiment, nearly all of which expressed detectable levels of Fgfr2 (Fig. 6h), were positive for cleaved Caspase-3 (Fig. 6i, j).

To overcome the problem of Fgfr2 perdurance in AT2 cells and corroborate these results, we employed two other approaches. In a classical pharmacologic approach (Fig. 6k), we instilled Fgfr inhibitor FIIN-1 into the lungs of adult (PN60) *Sftpc*^*CreER/+*^*; Rosa26*^*mTmG/+*^ mice treated with tamoxifen 2 weeks earlier to label mature AT2 cells. A fluorescent marker (WGA-405) was included in the instillation to identify regions of FIIN-1 exposure (Fig. 6l). As early as two hours after the instillation, inhibition of Fgfr signaling triggered widespread apoptosis of AT2 cells in the FIIN-1 exposed (WGA-405 labeled) regions (Fig. 6m, n), with no conversion to AT1 identity (Supplementary Fig. 7). To ensure this effect was attributable to Fgfr2 inhibition, we also tested a soluble recombinant Fgfr2b-Fc protein that binds its cognate ligands (see Fig. 1), preventing their engagement and activation of the receptor (Fig. 6o). With this inhibitor too, there was substantial induction of AT2 cell apoptosis in the exposed regions shortly (6 h) after instillation (Fig. 6p, q). Thus, acute inhibition of Fgfr2 or its ligands in the adult lung rapidly and robustly induces AT2 cell apoptosis.

Akt (Protein Kinase B), a serine-threonine kinase, functions downstream of Fgfr signaling to mediate cell survival in other contexts^41–43^. To determine if Akt signaling functions downstream of Fgfr2 signaling to mediate AT2 cell survival in adult mouse lungs, we activated Akt signaling by intraperitoneal injection of the small molecule Akt activator SC79 30 min prior to Fgfr2b inhibition by Fgfr2b-Fc installation. Activation of Akt almost completely abrogated AT2 apoptosis, as assayed by cleaved Caspase-3 (Supplementary Fig. 8). This suggests that Fgfr2 signaling promotes the survival of adult AT2 cells through activation of Akt.

### Loss of AT2 cells induces a robust regenerative response

**Fig. 7:**
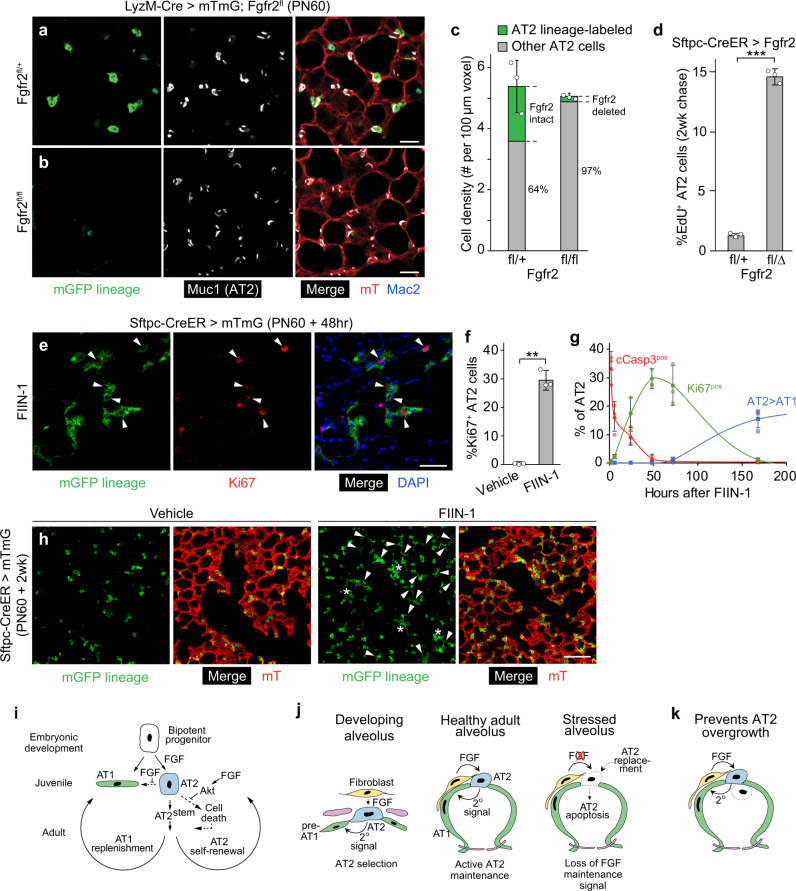
Robust regeneration following focal AT2 loss. **a**, **b** Alveolar regions of adult control *LyzM-Cre;Rosa26-mTmG;Fgfr2*^*fl/+*^ (**a**) and AT2 conditional deletion *LyzM-Cre;Rosa26-mTmG;Fgfr2*^*fl/fl*^ (**b**) mice immunostained as indicated. Note loss in **b** of lineage-labeled AT2 but preserved alveolar architecture. Mac2, macrophage marker. Bars, 50 µm. **c** Quantification of **a**, **b**. AT2 density is maintained in *Fgfr2*^*fl/fl*^ deletion by the compensatory expansion of unlabeled AT2 (with unrecombined *Fgfr*^*fl*^ alleles). *n* = 300 cells scored/condition (six animals, mean ± SD); *p* = 0.65 (not significant, Student’s two-sided *t*-test) for density difference. **d** AT2 proliferation in control adult *Sftpc-CreER;Rosa26-mTmG;Fgfr2*^*fl/+*^ and AT2 conditional *Fgfr2* deletion *Sftpc-CreER;Rosa26-mTmG;Fgfr2*^*fl/delta*^ by EdU labeling for 2 weeks following tamoxifen. *n* = 400 AT2 cells scored/condition (six lungs, mean ± SD). ***p = 5.4 × 10^−6^ (Student’s two-sided *t*-test). **e** Alveolar region of adult *Sftpc-CreER;Rosa26-mTmG* tamoxifen-induced to label AT2 and instilled with Fgfr inhibitor FIIN-1 then stained 48 h later for Ki67 and markers indicated. Arrowheads, proliferating AT2 (Ki67^pos^,GFP^pos^). Bar, 50 µm. **f** Quantification of **e**. *n* = 800 AT2 cells scored/condition (six animals, mean ± SD). **p = 2.6 × 10^−3^ (Student’s two-sided *t*-test). **g** Time course of AT2 apoptosis (cCasp3^pos^), compensatory AT2 proliferation (Ki67^pos^), and conversion to AT1 (Pdpn^pos^) after FIIN-1 instillation in adult *Sftpc*^*CreER/+*^*;Rosa26*^*mTmG/+*^ lungs (see Fig. 6k–n) and subsequent staining (AT2 lineage mGFP, cCasp3, Ki67, Pdpn). mGFP^pos^ (AT2 lineage) cells were scored in instilled (WGA^pos^) regions; uninstilled regions and vehicle-instilled lungs showed no changes. *n* = 300 mGFP^pos^ cells scored per stain/timepoint (21 animals, mean ± SD). **h** Vehicle and FIIN-1-treated alveolar regions 2 weeks after instillation. Note clusters of lineage-labeled AT2 (asterisks) and conversion to AT1 (arrowheads) in FIIN-1 lung, and overall restoration of the alveolar structure. Bar, 100 µm. Stain repeated in biological triplicate. **i** Sequential roles of Fgf/Fgfr2 pathway in alveolus: AT2 selection and 2^o^ induction of AT1 (embryo), AT2 fate consolidation and prevention of reprogramming to AT1 (juvenile), and AT2 survival (adult, mediated by Akt). AT2 loss triggers regeneration by AT2^stem^. **j** Source and dynamics of FGF signaling. After AT2 selection in development (left), a 2° signal induces other progenitors to AT1 fate. FGF signaling remains active in adults, maintaining AT2 (middle). Loss of FGF in the stressed alveolus (right) triggers AT2 apoptosis and rapid replacement. **k** FGF signaling in adults might prevent AT2 overgrowth/tumors by depriving daughters (dashed) moving away from the FGF source of survival signal. All experiments were repeated at least three times.

Despite the profound effects of Fgfr2 loss or inhibition on AT2 cells in juvenile and adult life, curiously, alveolar structure and the total number and proportion of AT2 cells were not grossly altered (Fig. 7a, b), obscuring its ubiquitous and constant requirement for AT2 cell maintenance throughout life. This is because the loss of targeted AT2 cells is almost completely compensated by the proliferation of untargeted cells. This was evident in the LyzM-Cre mice (*LyzM*^*cre/+*^*Fgfr2*^*fl/fl*^*;Rosa26*^*mTmG/+*^), where the population of untargeted AT2 cells (lacking the Cre-dependent lineage label) in PN60 mice was increased by 52% relative to that in the Fgfr2^+^ control *(LyzM*^*Cre/+*^*;Fgfr2*^*fl/+*^*;Rosa26*^*mTmG/+*^), maintaining the normal density of AT2 cells (~5 per 100 um^3^) and alveolar morphology despite the substantial loss of lineage-labeled AT2 cells (reduced from ~36% of AT2 cells in control *LyzM*^*Cre/+*^*;Fgfr2*^*fl/+*^*;Rosa26*^*mTmG/+*^ mice to 2.8% in *LyzM*^*cre/+*^*Fgfr2*^*fl/fl*^*;Rosa26*^*mTmG/+*^ mice) (Fig. 7c). The proliferative response of remaining AT2 cells following the loss of Fgfr2 activity in neighboring cells was apparent when loss was initiated by pharmacologic inhibition (Fig. 7e–g) or even following the inefficient deletion of *Fgfr2* in *Sftpc*^*CreER/+*^*; Fgfr2*^*del/fl*^ mice (Fig. 7d). The restorative response begins quickly because compensatory AT2 cell proliferation was detected by Ki67 immunostaining and EdU incorporation within hours of instillation of the Fgfr inhibitor FIIN-1 and initiation of AT2 apoptosis (Fig. 7f, g), and it continued for a week until alveolar structure was restored (Fig. 7g, h and Supplementary Fig. 9). The proliferating AT2 cells carrying out the restorative response (with Fgfr2 signaling intact because they were not from a FIIN-1-exposed area or because FIIN-1 had since cleared) are executing the canonical stem cell function of AT2 cells^5,44,45^, replacing lost AT2 cells and any AT1 cells lost along with them in the FIIN-1-exposed areas (Fig. 7h).

## Discussion

The results identify FGF signaling as the critical factor that induces and controls alveolar epithelial fate selection. *Fgfr2* is expressed in bipotent alveolar progenitors but rapidly restricts to nascent AT2 cells and remains exclusively in the AT2 lineage, while its ligands Fgf7 and Fgf10 are expressed in surrounding mesenchyme. The addition of either ligand to cultured progenitors induced the formation of alveolus-like structures with intermingled AT2 and AT1 cells, in the absence of any extrinsic forces or cell budding that might protect some progenitors from such forces. In mosaic cultures, randomly selected progenitors with constitutive *Fgfr2* expression exclusively acquired AT2 fate, whereas those with the Fgfr2 pathway inhibited exclusively acquired AT1 fate, and a similar result was obtained for *Fgfr2*^*-*^ progenitors in mosaic alveoli in vivo. The results demonstrate that Fgfr2 signaling serves as a developmental switch in alveolar development, inducing bipotent progenitors to AT2 fate (Fig. 7i); progenitors with no or low Fgfr2 signaling acquire AT1 fate through a secondary, cell non-autonomous signal provided by nascent AT2 cells. Recently, we identified a secondary signal (Notch pathway) required for AT1 fate selection (Gillich et al., in revision); we suspect this secondary signal could be enhanced by (or synergize with) some subsequent local mechanical change, which would integrate our findings with the longstanding models implicating mechanical forces in alveolar fate acquisition^18,23–25,46^.

The FGF developmental pathway remains on in AT2 cells throughout life. Loss of Fgfr2 signaling during the juvenile period results in AT2 reprogramming to AT1 fate, perhaps by direct reprogramming without proliferation^47^ or by transient reversion to bipotent progenitor identity (Supplementary Fig. 3d, e). Loss or reduction of Fgfr2 signaling during adult life, even briefly at any time or position in the lung, triggers rapid AT2 apoptosis, followed by a robust regenerative response (Fig. 7i). The anti-apoptotic effect of Fgfr2 signaling is transduced through Akt. Thus, the Fgfr2 pathway directly selects AT2 cells during development and then ubiquitously and continuously maintains them throughout life.

The Fgfr2 pathway also plays a critical role in the first six days of lung development, inducing and patterning airway branching^22,27,29,30,48^. The signaling pathway is thus reused throughout the life of the animal but with changing roles and cellular consequences at each stage. As the lung forms in the embryo (e10-e16), Fgfr2 signaling provides mitogenic and motogenic functions to pattern airway epithelium budding and branching. During late fetal life (e16-e19), its budding function may continue as it selects alveolar cell fates by directly inducing AT2 differentiation and triggering a secondary signal for AT1 fate. In early postnatal and juvenile life, it prevents AT2 reprogramming to AT1 fate, thereby consolidating the AT2 fate decision, whereas for the rest of life, it serves as a survival signal, continuously suppressing AT2 apoptosis likely through Akt. AT2 cells also require Fgfr2 during alveolar repair^32,36^. Although there has long been evidence of Fgfr2’s importance beyond airway branching^14,18,31–33,36,49,50^, its crucial functions in AT2 selection and lifelong maintenance have been obscured by the pathway’s earlier embryonic roles, by the difficulty in rapidly and completely deleting *Fgfr2* and depleting Fgfr2 protein, and by the robust regenerative response to alveolar cell loss by wild-type AT2 cells.

An important future goal will be to molecularly elucidate how the FGF signaling pathway selects and maintains AT2 cells, and how it results in distinct cellular outcomes at different stages in lung development. The same ligand (*Fgf7, Fgf10*) and receptor (*Fgfr2*) genes are deployed throughout life, so presumably, changes in the downstream signal transduction and effector pathway or in parallel pathways account for the stage-specific effects of Fgfr signaling. Such a model is operative in Drosophila respiratory system development where the initial round of Fgfr signaling induces changes in the expression of downstream effectors that alter the response in the next signaling round^51^. Appealing candidates for the differential apoptotic response of juvenile vs adult AT2 cells are anti-apoptotic BH3 proteins regulated by Akt activity^43,52^, which could buffer them against apoptosis. Other possible stage-specific effectors include ETV4 and ETV5, related transcription factors induced by Fgf7 and Fgf10 at bud tips during airway branching^53^ that regulate bud size, growth, and number^54^ but whose expression restricts such that ETV5 becomes specifically expressed in the AT2 lineage^11^, where it is required to maintain expression of AT2-selective genes in cultured cells as well as the adult mouse lung^55^.

The Fgfr2 pathway remains active in AT2 cells and sustains them throughout life (Fig. 7j). Why would a developmental signaling pathway remain on for months or even years after its developmental role has been completed, simply to keep the selected cell alive? The considerable energy expenditure involved in lifelong signaling suggests some substantial benefit of active AT2 maintenance. Perhaps it allows rapid conversion to a progenitor state and other fates following alveolar injury to quickly restore gas-exchange function, akin to the rapid transdifferentiation of club into ciliated airway cells when Notch signaling is blocked^47^. Or perhaps it prevents initiation of lung adenocarcinoma, the leading cancer killer, when an AT2 cell divides and a daughter loses contact with the underlying stromal cells that express the Fgf7 and Fgf10 survival signals (Fig. 7k). Whatever the reason, the pathway plays an important role in human lung health because genetic studies identify *Fgf7* as a susceptibility locus for the common and devastating disease COPD (chronic obstructive lung disease)^56^.

Our results show that loss or inhibition of Fgfr2 signaling has dire consequences for the alveolus and gas exchange, though they may not become apparent immediately because of the robust regenerative response activated by AT2 cell death. However, widespread targeting or loss of AT2 cells, as may occur in SARS, MERS, and COVID-19 coronavirus infections^6–8^, causes acute and severe alveolar injury and can be rapidly fatal. Pharmacologic modulation of Fgfr2 signaling could be used to support alveolar health during acute injuries like these, helping sustain AT2 cells as a virus or toxin destroys them^6–8^, and perhaps similarly in chronic diseases such as COPD/emphysema and pulmonary fibrosis^57^. Modulators could also be used to create alveolar cells for in vitro studies or cell therapies.

## Methods

### Mouse strains

All mouse experiments followed applicable regulations and guidelines and were approved by the Institutional Animal Care and Use Committee at Stanford University (Protocol 9780). Mice were housed under a 14 h light/10 h dark cycle or 12 light/12 dark cycle, and lights were not used during the dark cycle. Mouse housing temperature was kept at 65-75 °F (~18-23 °C) and humidity at 40–60%. Timed-pregnant C57BL/6 J females (abbreviated B6; Jackson Laboratories) were used for all embryonic time points, with gestational age verified by crown–rump length. For studies of adult wild-type lungs, B6 males and females were used. Mosaic labeling and deletion studies were conducted by Cre recombinase expression using gene-targeted alleles *BAC-Nkx2.1-Cre*, *LyzM* (also called *Lyz2*)*-Cre*, and *SftpC-Cre-ERT2-rtTA*^58^. Cre-dependent target genes were the conditional “floxed” *Fgfr2*^*fl/fl*^ for removal of *Fgfr2*, Cre reporter *Rosa26-mTmG*, which expresses membrane-targeted tdTomato (mT) in all tissues and mGFP following Cre-mediated recombination, and the Flp-dependent *Rosa26-MASTR* allele that constitutively expresses Cre recombinase following Flp recombination^37^. For initiating recombination with *SftpC-Cre-ERT2*, intraperitoneal injections of 3 mg tamoxifen were administered twice a week for 2 weeks, except where noted. Genomic DNA was extracted from tails by Proteinase K (Sigma) digestion and genotyping was performed by polymerase chain reaction (PCR) using published primer sets. Mice were housed in filtered cages and all experiments were performed in accordance with approved Institutional Animal Care and Use Committee protocols at Stanford University.

### Lung isolation and processing

For prenatal time points, individual embryos were staged by fetal crown–rump length before sacrifice and lungs removed en bloc. For postnatal time points, mice were euthanized by carbon dioxide inhalation and dissected to both expose the lungs and allow for exsanguination via the abdominal aorta. Phosphate buffered saline (PBS; Ca^2+^- and Mg^2+^-free, pH 7.4) was gently perfused into the right ventricle of the heart by syringe with a 21 gauge needle until the lungs appeared white. For postnatal time points at or after 8 days, the trachea was then cannulated with a blunt needle and the lungs were gently inflated to full capacity with molten low melting point agarose (Sigma, 2% in PBS). For all time points, lungs were then separated by lobe and fixed in either Zinc Formalin, 4% PFA, or Dent’s fixative at 4 ^o^C. For immunostaining, a vibrating microtome (Leica) was used to generate embryonic (200 µm) or adult (450 µm) tissue sections of uniform thickness. For in situ hybridization, lobes were submerged in OCT (Tissue Tek) in an embedding mold and frozen on dry ice. Tissue sections (10 µm) were obtained using a Leica CM3050S cryostat, collected on glass slides, and subsequently processed.

### Immunostaining

Immunohistochemistry was performed as previously described in ref. 59 using primary antibodies against the following epitopes (used at 1:500 dilution unless otherwise noted): pro-SftpC (rabbit, Chemicon AB3786), RAGE (rat, R&D MAB1179), E-cadherin (rat, Life Technologies ECCD-2), Podoplanin (hamster, DSHB 8.1.1), Mucin1 (hamster, Thermo Scientific HM1630 and rabbit, Novus NB120-15481), Ki67 (rat, DAKO M7249), Fgfr2 (rabbit, SCBT SC-122), cleaved Caspase-3 (rabbit, Novus NB100-56708), GFP (chicken, Abcam ab13970), Fgfr2iiib-Fc (human, R&D Systems) and Phospho-p44/42 MAPK (rabbit, CST D13.14.4E). Briefly, fixed lung slices were permeabilized and blocked overnight in 5% goat serum/PBS/0.5% Triton X-100 at 4 °C. Antibody incubation steps were conducted in a blocking buffer overnight and washing steps were performed in three rounds (15 min/round) using PBT (0.1% Tween-20/PBS). Primary antibodies were subsequently detected using Alexa Fluor-conjugated secondary antibodies (Life Technologies), including Goat anti-rabbit IgG Alexa 568 conjugated (Invitrogen, A11036), Goat anti-rat IgG Alexa 488 conjugated (Invitrogen, A11006), Goat anti-hamster Alexa 633 (Invitrogen, A-21113), and Goat anti-Human (Invitrogen, A-11014) unless noted otherwise and incubated in Vectashield with DAPI (5 µg/ml, Vector labs). EdU was detected using the standard Click-iT reaction (Thermo Fisher). Images were acquired using a laser-scanning confocal microscope (Zeiss LSM 780) and subsequently processed using Zen and ImageJ.

### In situ hybridization

Two methods of multiplexed single-molecule fluorescence in situ hybridization (smFISH) of mRNAs was performed. To simultaneously detect *Sftpc*, *Fgf7*, and *Fgf10* RNAs, RNAscope multiplex assay V1 kit (ACD) was used as described^11^. To visualize *Sftpc* and *Fgfr2* RNAs, proximity ligation in situ hybridization (PLISH) was used^60^. Briefly, 20 µm thick sections were cut from OCT-embedded, cryopreserved tissue and hybridized with multiple anti-sense probe pairs that hybridize to the target transcript. Probe pairs that targeted each gene share a common barcode that is unique and complementary to circle and bridge constructs. The circle and bridge constructs undergo proximity ligation to form a closed circle that undergoes rolling circle amplification. Detection oligonucleotides conjugated to fluorophores anneal to the rolling circle amplification product, generating discrete puncta for each transcript. The following sets of primer pairs were used to detect transcripts of the indicated genes:

#### Sftpc

5′ TCGTACGTCTAACTTACGTCGTTATGTGCGGTTTCTACCGACC3′

5′GGTCTTTCCTGTCCCGCTTATACGTCGAGTTGAAGAACAACCTG3′

5′TCGTACGTCTAACTTACGTCGTTATGTTTATTCTTTTGTGATAGGATCCC3′

5′TTGTTTTCCAATCAGGCTGCTTATACGTCGAGTTGAAGAACAACCTG3′

#### Fgfr2

5′TTAGTAGGCGAACTTACGTCGTTATGTCACCAGCGGGGTGTTGGAG3′

5′TTAGTAGGCGAACTTACGTCGTTATGTCATGTTTTAACACTGCCGTTTATGTGTGG3′

5′TTAGTAGGCGAACTTACGTCGTTATGAGACATGCTCATCGGACAGCAGAGT3′

5′TGTTGAGGACAGACGCGTTGTTATCCTTATACGTCGAGTTGAACATAAGTGCG3′

5′TGTTTGGGGACAGGAAGACACATTCACTTATACGTCGAGTTGAACATAAGTGCG3′

5′GTTTCTTGAAACATGGGCATTAGGGTGTCTTTATACGTCGAGTTGAACATAAGTGCG3′

Expression was detected by confocal fluorescence microscopy as ~0.5 µm fluorescent puncta.

### Cell isolation and culture

Adult AT2 cells and embryonic (e16.5) bipotent alveolar epithelial progenitors were purified as previously described in ref. 11. Adult 2-month-old mice were euthanized by administration of CO_2_. For e16.5, embryos were removed from the mother and their lungs were isolated en bloc without perfusion and pooled by litter (five to seven embryos) for further processing. Lungs were microdissected to remove the proximal lung tissue, leaving only the distal (alveolar region) tissue. Distal e16.5 lung cells were dissociated in dispase (BD Biosciences) and triturated with glass Pasteur pipettes until a single-cell suspension was attained. For adult lungs, the vasculature was perfused through the right ventricle with 37 °C media (DMEM/F12, Life Technologies). The trachea was punctured, and lungs were inflated with digestion buffer (DMEM/F12 containing elastase (1 U/ml, Worthington) and dextran (10%, Sigma)) for 20 min at 37 °C. Digested lungs were minced with a razor blade into 1 mm^3^ fragment, suspended in 5 ml of digestion buffer containing DNase I (0.33 U/ml; Roche), incubated with frequent agitation at 37 °C for 45 min, and triturated briefly with a 5-ml pipette.

To deplete red blood cells, an equal volume of DMEM/F12 supplemented with 10% FBS and penicillin–streptomycin (1 U/ml; Thermo Scientific) was added to the lung single-cell suspensions before filtering through 100 µm mesh (Fisher) and centrifuging at 400×*g* for 10 min. Pelleted cells were resuspended in red blood cell lysis buffer (BD Biosciences), incubated for 2 min, passed through a 40 µm mesh filter (Fisher), centrifuged at 400×*g* for 10 min, and then resuspended in MACS buffer (2 mM EDTA, 0.5% BSA in PBS, filtered and degassed) for purification.

From the resultant single-cell suspensions of distal e16.5 lung, bipotent progenitors were isolated by MACS using MS columns (Miltenyi Biotec) according to the vendor protocol. Before column loading, suspensions were passed through a 35 µm cell strainer (BD Biosciences). Other cell types were first depleted with antibodies against CD31, CD45, and Pdgfrα (Miltenyi Biotec), then bipotent progenitors were positively selected using a biotinylated EpCAM antibody (clone G8.8, eBiosciences, 1:100 dilution) and streptavidin-conjugated magnetic beads (Miltenyi Biotec). This generates a highly enriched preparation of e16.5 bipotent alveolar progenitors (94 ± 4.6% Sftpc^+^ Rage^+^ cells, *n* = 416 purified cells scored across three biological replicates^5^). Adult AT2 cells were isolated from single-cell suspensions of adult lungs in the same way^61,62^, generating a highly enriched preparation of AT2 cells (92% GFP^+^ AT2 cells, as shown by genetic labeling with Sftpc-CreER;mTmG, *n* > 200,000 purified cells analyzed by flow cytometry in experimental duplicate). To enrich for adult fibroblasts, MACS depletion was conducted as above using antibodies against CD31, CD45, and EpCAM.

For culturing bipotent progenitors, cell density was calculated using a hemocytometer. A density of 100,000 cells per well was used for culture in eight-well #1 coverglass chambers (Labtek) precoated with growth factor reduced Matrigel (80 µl, BD Biosciences) for 30 min at 37 °C. Cells were supplemented with Fgf7 (50 ng/ml, R&D Systems), Fgf10 (100 ng/ml, R&D Systems), FIIN-1 (20 nM, Tocris), Fgfr2iiib-Fc (1 µg/ml, R&D Systems), Arp2/3 inhibitor CK666 (40 µM, R&D Systems), ROCK inhibitor Y27632 (20 µM, R&D Systems), and heparan sulfate proteoglycans (HSPG, 100 ng/ml, Sigma) to the indicated concentrations in DMEM/F12. Cells were maintained at 37 °C in 400 µl of DMEM/F12 with media changes every other day in a 5% CO_2_/air incubator, typically for 4 days, except where indicated otherwise.

### Live imaging of alveolar progenitors in culture

Time-lapse microscopy of cultured alveolospheres was conducted using an inverted Zeiss LSM 880 confocal microscope equipped with environmental control to maintain 37 °C and 5% CO_2_. Isolated bipotent progenitors were plated and cultured for ~12 h before confocal microscopy. Brightfield z-stacks (20x multi-immersion objective, NA 1.33) were acquired every 15 min for at least 3 days. The lumenal area, measured at the center z-position of each organoid, was used as an indicator of stretch, as commonly implemented in forskolin-induced swelling experiments^63^. The budding status of a cell was determined using the published criteria^18^ of basal extrusion with a substantial reduction of the lumenal cell surface.

### Single-cell RNA sequencing

Processing, scRNAseq, and analysis of e18.5 and adult mouse lung cells was conducted as previously described with minor alterations to enrich the respective populations^11^. Briefly, single-cell suspensions sorted by MACS as CD45^−^ CD31^−^ EpCAM^-^ (for mesenchymal cells) or CD45^−^ CD31^+^ EpCAM^−^ (for endothelial cells) were subsequently incubated with a viability stain (Sytox Blue, Life Technologies) for 15 min and loaded on a medium-sized (10–17 µm cell diameter) microfluidic RNA-seq chip (Fluidigm) using the Fluidigm C1 system. Cells were loaded at a concentration of 300–500 cells/ml and imaged by phase-contrast and fluorescence microscopy to assess the number and viability of cells per capture site. Only single, live cells were included in the analysis. For scRNAseq experiments, cDNAs were prepared on a chip using the SMARTer Ultra Low RNA kit for Illumina (Clontech). ERCC (External RNA Controls Consortium) RNAspike-in Mix (Ambion, Life Technologies) was added to the lysis reaction and processed in parallel to cellular messenger RNA. Sequencing libraries were constructed (Illumina Nextera XT DNA Sample Preparation kit), and single-cell libraries pooled and sequenced 100 base pairs (bp) paired-end to a depth of 2 to 6 × 10^6^ reads. Sequencing data were processed as described in ref. 11, and transcript levels quantified as fragments per kilobase of transcript per million mapped reads (FPKM) generated by TopHat/Cufflinks. Cells not expressing (FPKM <1) three of four housekeeping genes (*Actb*, *Gapdh*, *Ubc*, *Ppia*), or expressing them less than three standard deviations below the mean, were removed from the analysis. Fibroblasts were identified as cells expressing canonical fibroblast markers *Mgp*, *Col1a1*, and *Col1a2* that also lacked expression of canonical markers of the major airway or alveolar epithelial cell types. Subsequent analysis, including hierarchical clustering, was performed as described in ref. 11. To determine isoform expression of *Fgfr2*, STAR was used to align and count reads specific to either exon IIIb or IIIc To determine receptor genes restrictively expressed along either AT1 or AT2 lineage, receptor genes were first screened for ones detectably expressed in at least six cells, then Welch’s *t*-test was used to identify receptor genes with at least twofold higher expression in each respective lineage (*p* ≤ 0.01).

For the analysis of cell type-specific gene expression in published scRNAseq datasets, the processed mRNA counts for each cell were used from developing (SE119228)^10^ and adult mouse lung (GSE109774)^64^ datasets. Expression matrices were analyzed via R using the Seurat package. Clusters of cells with similar expression profiles were identified via shared nearest neighbor analysis using the Louvain algorithm, clusters were visualized in UMAP plots, and expression of specific genes was represented in heatmaps, violin plots, and dot plots generated in Seurat.

### Mosaic and inducible deletion of *Fgfr2*

To mosaically modulate Fgfr2 activity in culture, a lentiviral approach was developed using the Lenti-X Tet-On 3 G Inducible Expression System (Clontech). The ORF encoding Fgfr2iiib (Origene; MC221076), the IRES sequence from pLVX-IRES-Puro (Clontech; 632186), and the sfGFP (superfolder GFP) coding sequence from pBAD-sfGFP (Addgene; 54519) were PCR-amplified with adapter sequences (IDT) and assembled (NEB; E5520) into the lentiviral backbone pTetOne (Takara; 631018) linearized by EcoRI/BamHI digestion (NEB) to generate pTetOne-Fgfr2-IRES-sfGFP (Fig. 3a). To generate a dominant negative form of Fgfr2iiib, a truncated version of Fgfr2iiib lacking the tyrosine kinase domain^65^ was also PCR-amplified with adapter sequences (as above) to assemble pTetOne-Fgfr2^DN^-IRES-sfGFP. Lentiviruses (“Lenti-Fgfr2”, “Lenti-Fgfr2^DN^”) were produced from these plasmids following the manufacturer’s instructions for transfection, lentiviral concentration, and titer estimation (Lenti-X Expression System, Clontech). An AAV virus that constitutively expresses eGFP from CMV promoter (Addgene, 105545-AAV9) was used as a cell labeling control (Supplementary Fig. 4b). Purified bipotent progenitors plated on Matrigel in a standard medium as above were treated with cationic polymer polybrene (8 ng/µl, EMD Millipore, to enhance viral infection) 10 min before viral addition. After 24 h of infection at 37 ^o^C in media containing doxycycline (100 ng/ml, Sigma) to induce expression of the Fgfr2iiib genes, media was replaced with media containing doxycycline and Fgf7 (50 ng/ml). Four days later, cultured cells were fixed and analyzed by immunostaining.

For in vivo mosaic labeling and deletion of the floxed *Fgfr2* allele in bipotent progenitors, *Tg*^*Nkx2.1-Cre*^; *Rosa26*^mTmG/mTmG^ mice were used. For analogous experiments in AT2 cells, *LyzM*^*Cre*^; *Rosa26*^mTmG/mTmG^ and *Sftpc*^*Cre-ERT2-rtTA*^; *Rosa26*^mTmG/mTmG^ were bred and induced by intraperitoneal injection of tamoxifen. At time points and conditions indicated, lineage-labeled (mGFP+) and unlabeled (mGFP−) cell types (BP, AT2, or AT1, as indicated) identified by marker expression were counted from large alveolar fields (25x objective, ~500 µm × 500 µm × 100 µm).

To mosaically but efficiently delete *Fgfr2* in adult AT2 cells, we used a recombinant AAV described below to infect and express Flp recombinase mosaically in AT2 cells, and the Flp-dependent *Rosa26-MASTR* allele to constitutively express Cre recombinase^37^ to delete the conditional *Fgfr2*^*fl/fl*^ allele. The pAAV-EF1α-Flpo plasmid^66^ was modified using the assembly scheme described above (NEB; E5520) to replace the EF1α promoter (removed by MluI/BamHI restriction digestion) with a previously defined^38^ 320 bp minimal promoter element of *Sftpc* that was chemically synthesized with added adapter sequences (IDT) (Fig. 6c). Recombinant AAV-Sftpc-Flpo virus was prepared by the HHMI Janelia Farm Viral Tools facility (2 × 10^14^ GC (genome copies)/ml, serotype 2/9) and endotracheally instilled (1 µl of viral solution diluted in 50 µl PBS) into the lungs of mice harboring *Rosa26*^*MASTR/mTmG*^ and either heterozygous or homozygous for the floxed *Fgfr2* allele. The efficiency of *Fgfr2* deletion was assessed by immunostaining for Fgfr2. For adult time points, the density of AT2 cells was calculated and expressed as the number of AT2 cells per 100 µm × 100 µm × 100 µm region of alveolar tissue. To determine AT2 proliferation, EdU (1 mg/ml, Cedar Lane) was administered in the drinking water for the indicated period.

### Pharmacologic inhibition of Fgfr2 and activation of Akt

To rapidly and persistently inhibit Fgfr2 signaling, the irreversible Fgfr2 inhibitor FIIN-1 (14.75 mg/kg, Tocris)^67–69^ or recombinant Fgfr2iiib-Fc (100 µg/30 g mouse, R&D Systems) was used. Mice at least 2 months (and up to over 1 year) of age carrying the Sftpc^Cre-ERT2-rtTA^; Rosa26^mTmG/mTmG^ alleles were administered tamoxifen (3 mg/30 g mouse, Sigma) via intraperitoneal injection to label >95% of AT2 cells. After 1 week, the mice were anesthetized with isoflurane and administered by endotracheal instillation either vehicle alone (PBS with biotinylated WGA), or vehicle with the inhibitor. To test the effect of Akt signaling, the small molecule Akt activator SC79 (Tocris) was administered (0.04 mg drug per g mouse) via intraperitoneal injection 30 min prior to Fgfr2 inhibition. Lungs were isolated at the indicated time points and areas of high exposure to inhibitors were identified by WGA immunostaining.

### Statistical analysis

Data analysis and statistical tests were performed with R or GraphPad Prism software. Replicate experiments were all biological replicates with different animals, and quantitative values are presented as mean ± SD unless indicated otherwise. Student’s *t*-tests were two-sided. No statistical method was used to predetermine sample size, and data distribution was tested for normality prior to statistical analysis and plotting. Both male and female animals were used in experiments, and subjects were age- and gender-matched in biological replicates and in comparisons of different groups. Post hoc power analysis was done (Supplemental Table 1).

### Reporting summary

Further information on research design is available in the Nature Research Reporting Summary linked to this article.

## Supplementary information

Supplementary Information

Reporting Summary

## Data Availability

The scRNAseq datasets are available in the GEO repository under the accession numbers “GSE109774”, “GSE52583” for E18 distal lung epithelial cells, “GSE196874” for E18 distal lung mesenchymal and endothelial cells, “GSE109444” for adult lung mesenchymal cells, and “GSE119228” for E16 lung cells. Source data are provided with this paper.

## Source data

Source Data

## Code availability

Code to reproduce the analyses described in this manuscript can be accessed at https://github.com/dbrownfield/Brownfield_et_al, 451970378.
