# Supplementary material for: Alveolar cell fate selection and lifelong maintenance of AT2 cells by FGF signaling
Source: Nat Commun. 2022 Nov 21;13:7137. doi: 10.1038/s41467-022-34059-1 (PMC9681748; doi:10.1038/s41467-022-34059-1)
Supplement: Supplementary file 1 — Supplementary Information [file 41467_2022_34059_MOESM1_ESM.pdf]

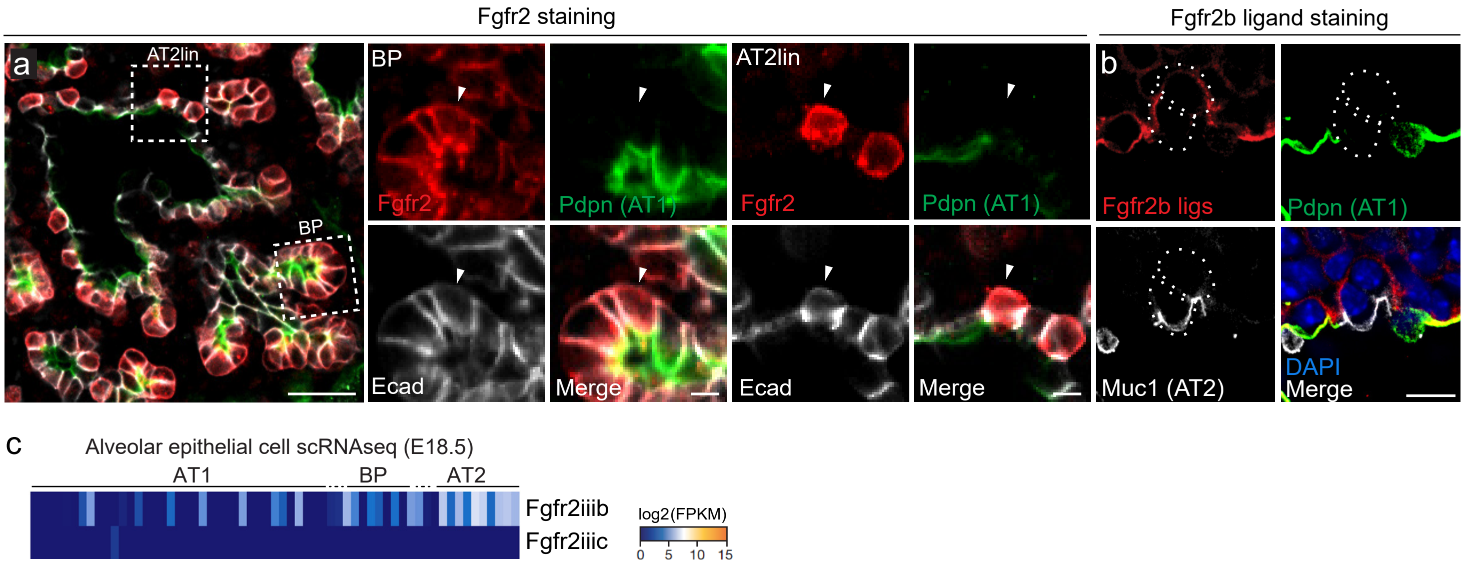

Supplementary Figure 1. Additional examples of expression of Fgfr2 and its ligands during alveolar differentiation. (a) e17.5 lung immunostained for Fgfr2, AT1 marker podoplanin (Pdpn), and epithelial marker E-cadherin (E-cad) as in Fig. 1c. Boxed regions, close-ups and split channels at right of bipotent progenitor (BP) and developing AT2 lineage cell (AT2 lin). Note developing AT2 lineage cell (arrowhead) has lost expression of Pdpn so is more advanced in development than one shown in Fig. 1c that still expresses it. Bars, 50µm (left panel) and 10µm (right panels). (b) e17.5 lung stained with the Fgfr2 (isoform iiib) ligand-binding domain fused to human IgG_1 domain as in Fig. 1d to show Fgfr2b ligands, and co-stained for Pdpn, and AT2 marker mucin1 (Muc1). Note two adjacent developing AT2 cells (dotted circles) and diffuse distribution of Fgfr2b ligands surrounding their basal surface and that of neighboring AT1 cells. Bar, 10µm. Stain repeated in biological triplicate. (c) scRNAseq heatmap of E18.5 alveolar epithelium as in Fig. 1a except Fgfr2 transcripts specific to Fgfr2iiib and Fgfr2iiic isoforms were mapped and calculated separately. Note Fgfr2iiib is almost exclusively expressed. All experiments were repeated at least three times.

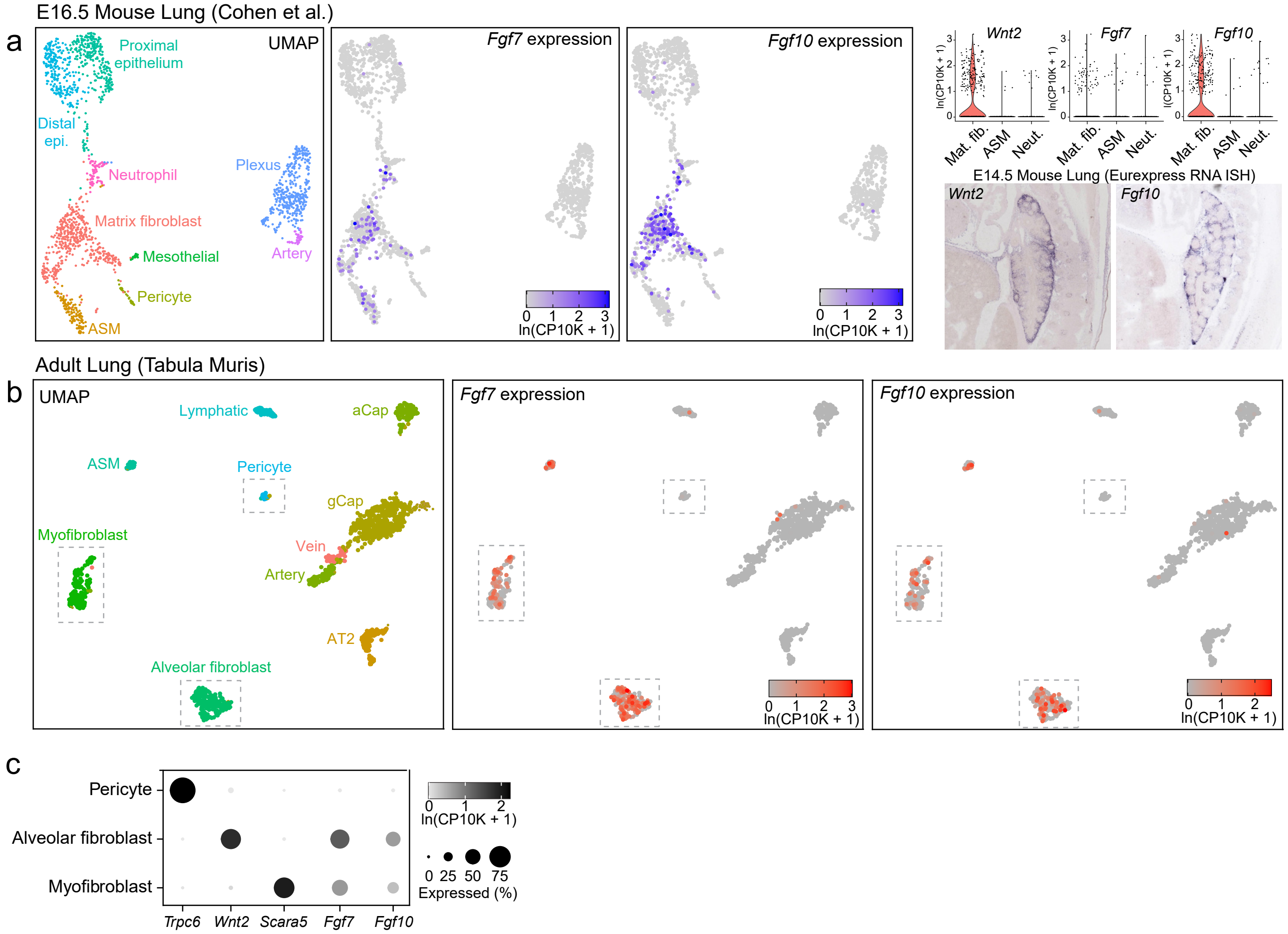

Supplementary Figure 2. Identification by scRNAseq of the mesenchymal Fgf ligand source in the developing and adult mouse lung. (a) UMAP of transcriptomic profiles of e16.5 mouse lung cells[1] clustered via Louvain algorithm and cell clusters identified as matrix fibroblast (*Mfap4, Wnt2*), airway smooth muscle (ASM, *Enpp2*), pericyte (*Gucy1a3*), mesothelial (*Wt1*), neutrophil (*Retnlg*), distal epithelial (*Sox9*), proximal epithelial (*Sox2*), capillary plexus (*Aplnr*), and arterial (*Gja5*) cell types by canonical marker gene expression[1, 2, 3 ,4] (Fib., fibroblast; Epi., epithelial). Gene expression values in heatmaps and violin plots were normalized, scaled and and log-transformed using Seurat (https://satijalab.org/seurat/) and given as ln(CP10K + 1)). Heatmaps (left) and violin plots (upper right) for *Fgf10* and *Fgf7* show both are expressed predominantly in matrix fibroblasts (Mat. fib., *Wnt2*-expressing) with sparse expression in airway smooth muscle (ASM) and neutrophils (Neut). (Lower right) RNA in situ hybridization of lung sections from e14.5 embryos (Eurexpress) show similar spatial expression patterns of *Wnt2* and *Fgf10*. (b) UMAP of transcriptomic profiles of adult mouse lung cells clustered via Louvain algorithm and cell clusters identified by canonical marker expression (ASM, *Acta2*; myofibroblast (Myofib), *Scara5*; alveolar fibroblast (Alv. Fib) , *Wnt2*; pericyte, *Trpc6*; lymphatic endothelium (Lymph), *Gja1*; aerocyte capillary (aCap), *Apln*; general capillary (gCap), *Aplnr*; artery/venous endothelium (Art/Vein), *Vwf*; AT2, *Sftpc*)[2, 3, 4] . Among the alveolar mesenchymal cell types (dashed boxes), note expression of both *Fgf7* and *Fgf10* in alveolar fibroblasts and, to a lesser extent, in myofibroblasts but not in pericytes. (c) Dotplot showing quantification (dot intensity, expression level; dot size, percent of cells with expression > 0) of *Fgf7* and *Fgf10* expression values from panel B along with selected cell type marker genes.

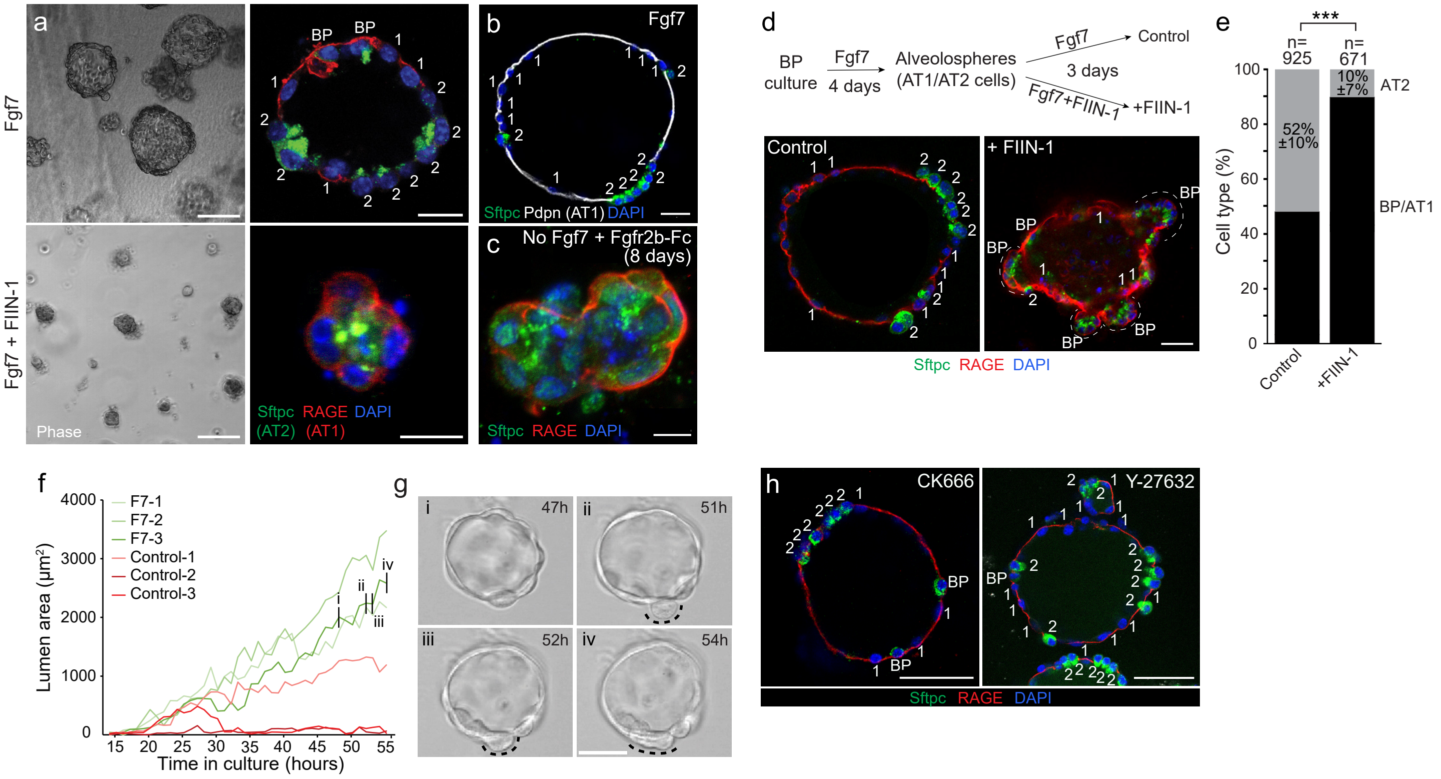

Supplementary Figure 3. Live imaging of alveolosphere development in culture and effects of Fgfr and Arp2/3 inhibitors. (a) Phase images (left) and immunostains (right) of epithelial progenitors purified from tips of e16.5 lungs and cultured in Matrigel as in Fig. 2 for four days with Fgf7 alone (50 ng/ml added every two days; upper panels) or with Fgfr inhibitor FIIN-1 (10 nM; bottom panels). Note that Fgfr inhibition prevents bipotent progenitors (co-express Sftpc and RAGE) from forming alveolospheres (left) with differentiated AT1 and AT2 cells (right, same image as Fig. 2). BP, bipotent progenitor; 1, AT1 cell; 2, AT2 cell. Bars, 20µm. Stain repeated in biological triplicate. (b) Immunostaining of alveolospheres cultured with Fgf7 for 4 days and stained for the AT1-specific marker Podoplanin (white). Note expression is restricted to flat, Sftpcneg AT1 cells. Bar, 20µm. Stain repeated in biological triplicate. (c) Culture of e16.5 progenitors for 8 days in culture without FGF addition and with Fgfriiib-Fc (1µg/ml) added to block any FGF ligands provided by growth-factor-reduced Matrigel. Note progenitors remain undifferentiated bipotent progenitors (BP; $Sftpc^{+}$ $RAGE^{+}$) even after this extended culture. Bar, 10µm. Stain repeated in biological triplicate. (d) Alveolospheres generated by culturing progenitors for 4 days as in a and then cultured an additional three days in the continued presence of Fgf7 (50ng/ml) alone as control (left) or with added FIIN-1 (right, 10nM). Note loss of AT2 cells ($Sftpc^{+}$ $Rage^{-}$, labeled "2") and concomitant increase in AT1 cells ($Sftpc^{-}$ $RAGE^{+}$, labeled "1") and bipotent progenitors ($Sftpc^{+}$ $RAGE^{+}$, labeled "BP"). Bar, 20µm. (e) Quantification of panel d for AT2 cells ($Sftpc^{+}$ $RAGE^{-}$) and BP/AT1 cells ($Sftpc^{+}$ $RAGE^{+}$ and $Sftpc^{-}$ $RAGE^{-}$ cells). n, number of cells scored for each condition in experimental triplicate (mean +/- SD). ***, $p=1.2x10^{-6}$ (Student's two-sided t-test). (f) Quantification of maximum luminal area in timelapse videomicroscopy recording of individual alveolospheres in control cultures without Fgf7 (red, Control-1, -2, -3) or with added Fgf7 (green, F7-1, F7-2, F7-3) as in a. Note continuous growth of all three alve-olospheres in the cultures with Fgf7. (g) Frames at the indicated times from the alveolosphere F7-3 culture in panel f showing a transient cell budding (dashed arc) at hour 51, which soon regresses (hours 52, 54). Note luminal expansion has occurred (panel f) and AT1 flattening is already apparent at hour 47. Bar, 20µm. (h) Alveolosphere cultured in the presence of Fgf7 (50ng/ml) and either the Arp2/3 inhibitor CK666 (40µM) or the ROCK inhibitor Y27632 (20µM). AT2 and AT1 cells appear to develop normally, despite a reduction in transient budding events (with CK666 treatment) noted in live imaging (not shown). Bar, 20µm. All experiments were repeated at least three times.

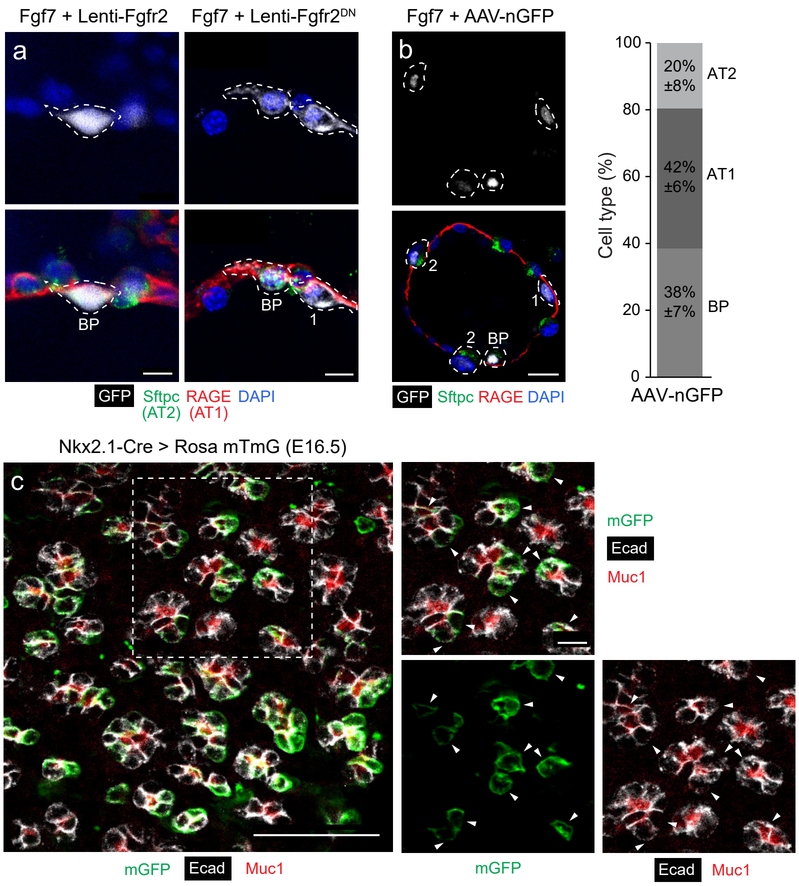

Supplementary Figure 4. Mosaic cell labeling of alveolar cells in culture and in vivo.
(a) Examples of the rare lentivirus-infected (GFP$^+$) cells (dashed outlines) from Fgf7-induced alveolospheres in Fig. 3 that remained as a bipotent progenitor (BP, Sftpc$^+$ RAGE$^+$) despite infection with the indicated Fgfr2 lentivirus that caused nearly all infected cells to develop as AT2 cells (Lenti-Fgfr2, left panel) or AT1 cells (Lenti-Fgfr2^{DN}, right panel; "1", Sftpc$^-$ Rage$^+$) (see Fig. 3). Bars, 10µm. Stain repeated in biological triplicate. (b) Control alveolosphere as in Fig. 3 except treated instead with AAV-nGFP prior to Fgf7 induction, then immunostained for indicated markers to determine cell identity (BP, AT1, AT2, left panel) acquired during culturing. Bar, 20µm. Quantification of acquired identities of infected (GFP$^+$) cells (right panel, n=50 GFP$^+$ cells scored in experimental triplicate) shows that progenitors infected with this control virus (lacking Fgfr2) have a similar chance of acquiring either AT1 (42 +/-6%, mean +/- SD) or AT2 (38 +/-7%) fate. (c) Distal epithelium of e16.5 lung from control Nkx2.1-Cre; Rosa26-mTmG mouse (similar to Fig. 4) immunostained for mGFP, E-cadherin and Muc1, which labels bipotent progenitors at this stage. Right panels show close up (with split channels as indicated) of boxed region in left panel. Note sporadic mGFP$^+$ cells (arrowheads) intermixed with mGFP$^-$ cells in epithelium, showing Nkx2.1-Cre activity in distal epithelium is highly mosaic rather than uniform. Bars 50µm, 10µm (insets). Stain repeated in biological triplicate. All experiments were repeated at least three times.

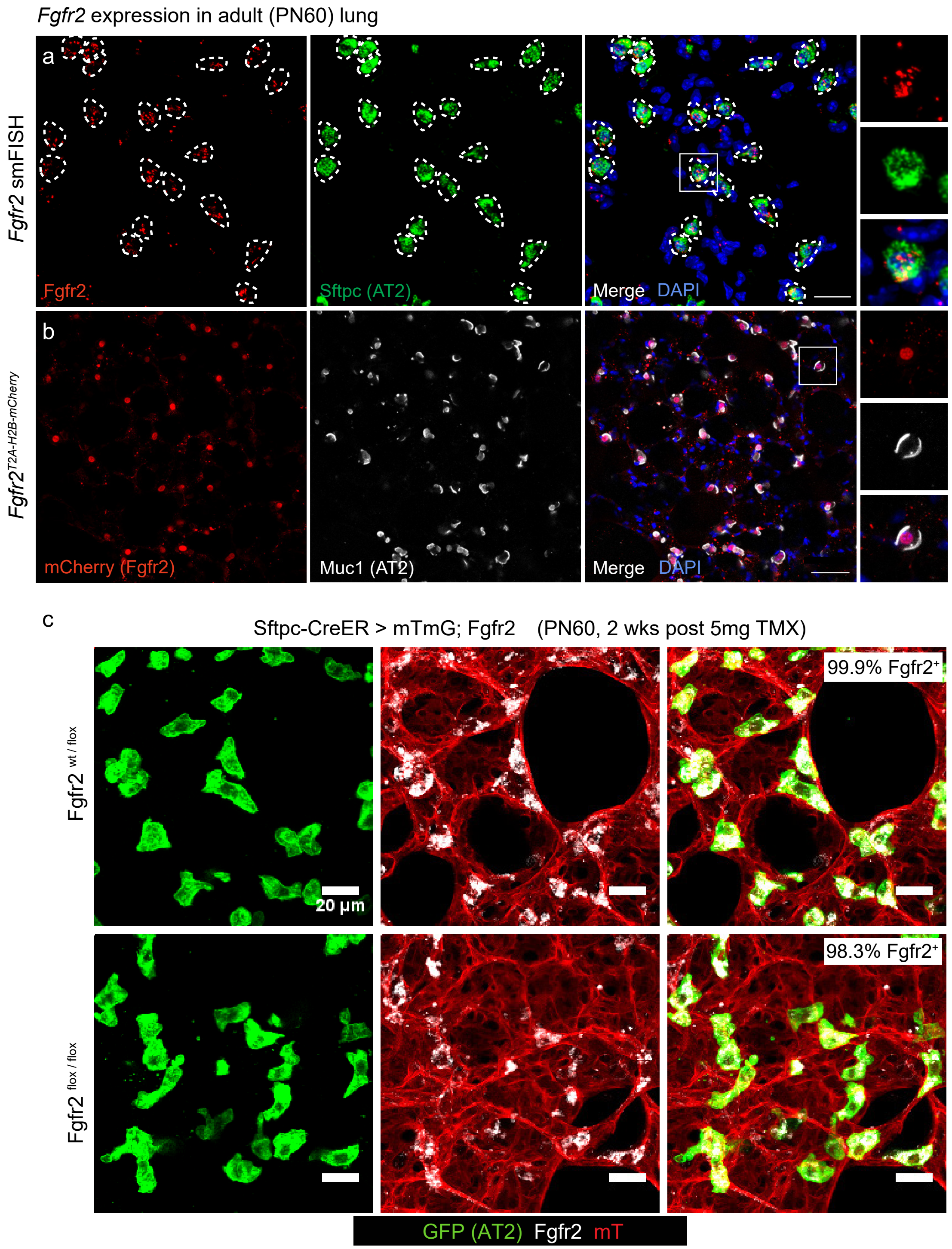

Supplementary Figure 5. *Fgfr2* is selectively expressed in adult AT2 cells.
(a) Single molecule fluorescence in situ hybridization (smFISH; PLISH) of alveolar region of adult (PN60) mouse lung for *Fgfr2* (red) and AT2 cell marker *Sftpc* (green). Right panels, close-up of boxed region. Note co-expression of *Fgfr2* and *Sftpc* mRNA indicating only AT2 cells express *Fgfr2*. Bar, 20µm. Stain repeated in biological triplicate. (b) Immunostain of alveolar region of adult (PN60) *Fgfr2*$^{T2A\text{-}H2B\text{-}mCherry}$ lung, carrying an *Fgfr2* knock-in reporter allele, immunostained for mCherry reporter and AT2 marker Muc1. Note selective expression of *Fgfr2* reporter in AT2 cells. Bar, 50µm. Stain repeated in biological triplicate. (c) Immunostain of alveolar regions of adult (PN60) *SftpC-CreER; Rosa26-mTmG; Fgfr2*$^{fl/+}$ (upper panels) and *SftpC-CreER; Rosa26-mTmG; Fgfr2*$^{fl/fl}$ (lower panels) two weeks following tamoxifen (5mg) induction of CreER to label AT2 cells with GFP and delete *Fgfr2*^{fl}. Note that despite the high tamoxifen dose to activate CreER, Fgfr2 protein is still detected (white) in virtually all AT2 cells (>98% of GFP^{+}) Bar, 20µm. Stain repeated in biological triplicate. All experiments were repeated at least three times.

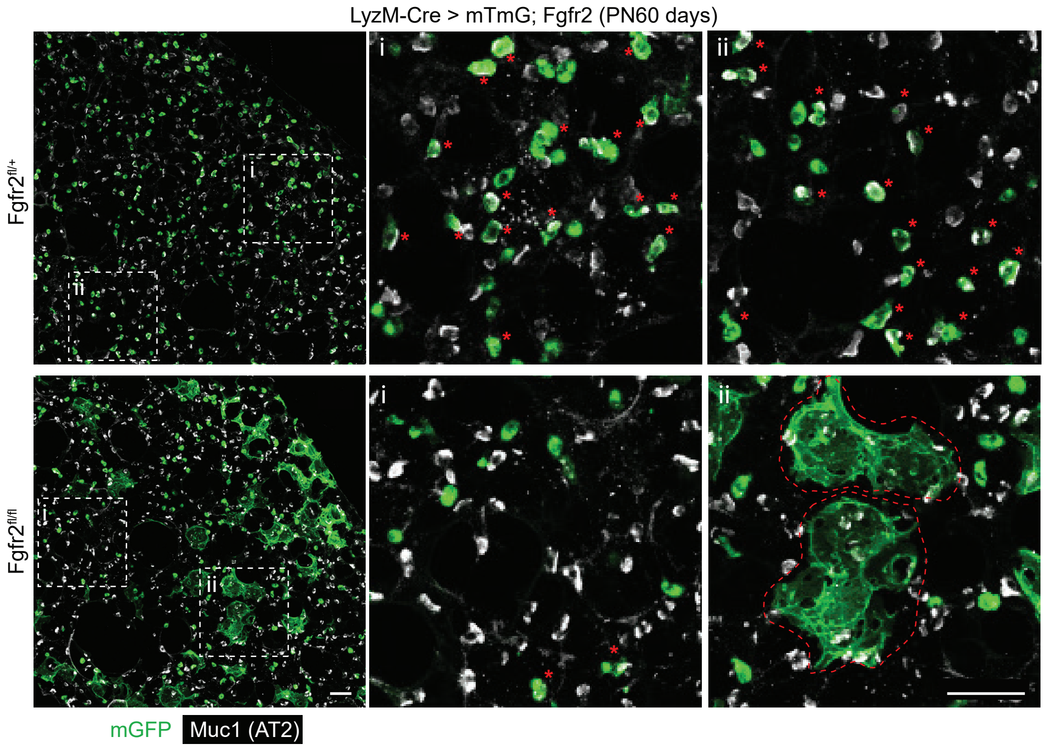

Supplementary Figure 6. Large field of view of adult lungs following mosaic *Fgfr2* deletion in AT2 cells throughout life. Tiled maximal intensity projections showing large field of view of alveolar regions of adult (PN60) control Fgfr2^{+} *LyzM-Cre; Rosa26-mTmG; Fgfr2$^{fl/+}$* (upper panels) and AT2 cell conditional deletion *LyzM-Cre; Rosa26-mTmG; Fgfr2$^{fl/fl}$* (lower panels) mice (see Fig. 7a,b) immunostained for markers indicated. Although there are many regions in lung in lower panel (e.g. box i) where GFP lineage-labeled Muc1^{pos} AT2 cells (red asterisks) are depleted due to Fgfr2 deletion as in Fig. 7b (the other GFP lineage-labeled cells are alveolar macrophages, which lack Muc1), note there are also scattered mGFP lineage-labeled AT1 cells in this lung (box ii, lower panel; large cup-shaped cells, red dashed outlines), which presumably persist after their generation by *Fgfr2* loss from AT2 cells during the perinatal period. Bars, 50µm. Stain repeated in biological triplicate.

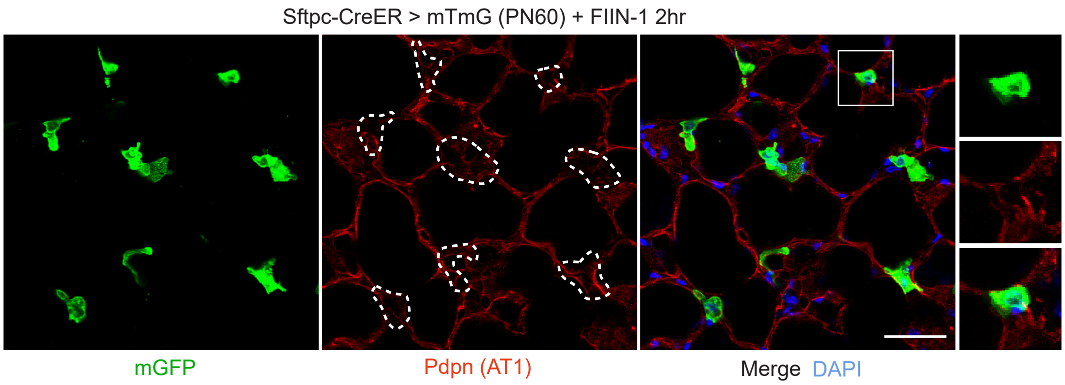

Supplementary Figure 7. Fgfr inhibition in adult lung does not cause early reprogramming to AT1 fate. Alveolar region of adult (PN60) *Sftpc-CreER; Rosa26-mTmG* lung induced with tamoxifen to label AT2 cells with GFP, then one week later instilled with Fgfr inhibitor FIIN-1 as in Fig. 6c-f and stained 2 hours later for GFP and AT1 marker Podoplanin (Pdpn) with DAPI counterstain. Right panels, close-up of boxed region. Although many lineage-labeled (GFP^{pos}) AT2 cells in exposed region are undergoing apoptosis at this time (see Fig. 6e,f), note that none of the lineage-labeled cells (dashed outlines; 0 of 398 GFP^{pos} cells scored in 3 experiments) showed evidence of reprogramming to AT1 fate (squamous morphology or expression of AT1 marker Pdpn). Bar, 50µm.

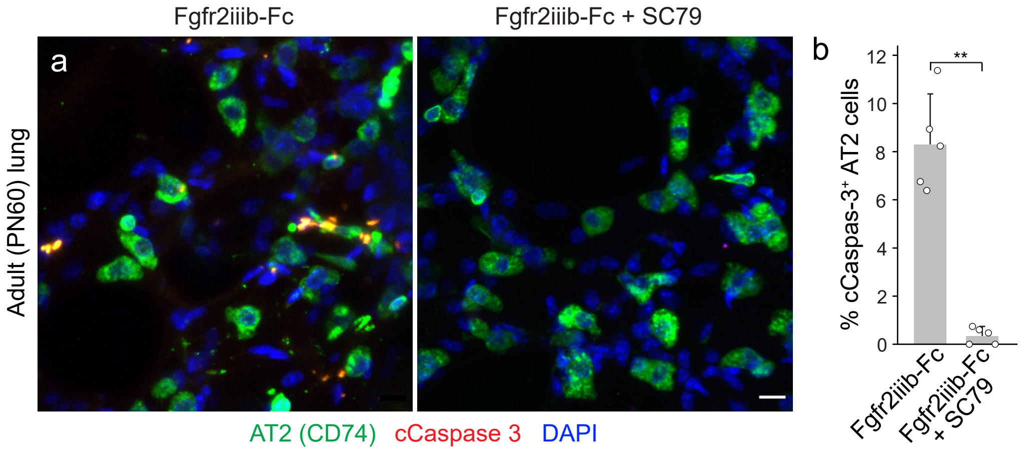

Supplementary Figure 8. AT2 cell death following Fgfr2 inhibition is abrogated by Akt activation. (a) Close-ups of WGA-labeled alveolar regions from adult (PN60) lungs co-stained for AT2 marker CD74 and apoptotic marker cleaved Caspase 3 (cCaspase 3) and counterstained with DAPI 6 hours after the lungs were co-instilled with WGA-405 and Fgfr2iiib-Fc (as in Fig. 6o,p) without a pre-treatment as control (left) or with pre-treatment (intraperitoneal injection 30 min before WGA-405/Fgfr2iiib-Fc instillation) with Akt activator SC79 (0.04 mg/g mouse) (right). Note AT2 cell apoptosis (cCaspase 3 expression) following inhibition of Fgfr2 by Fgfr2iiib-FcAT2 (left) is prevented by Akt activation with SC79 (right). Bars, 10µm. (b) Quantification of a showing percent of AT2 cells that are apoptotic (cCaspase 3^+) in control and SC79-pre-treated lungs. n=120 AT2 cells scored per condition in experimental triplicate (mean +/- SD); **, p-value $=1.3\times10^{-3}$ (Student's two-sided t-test).

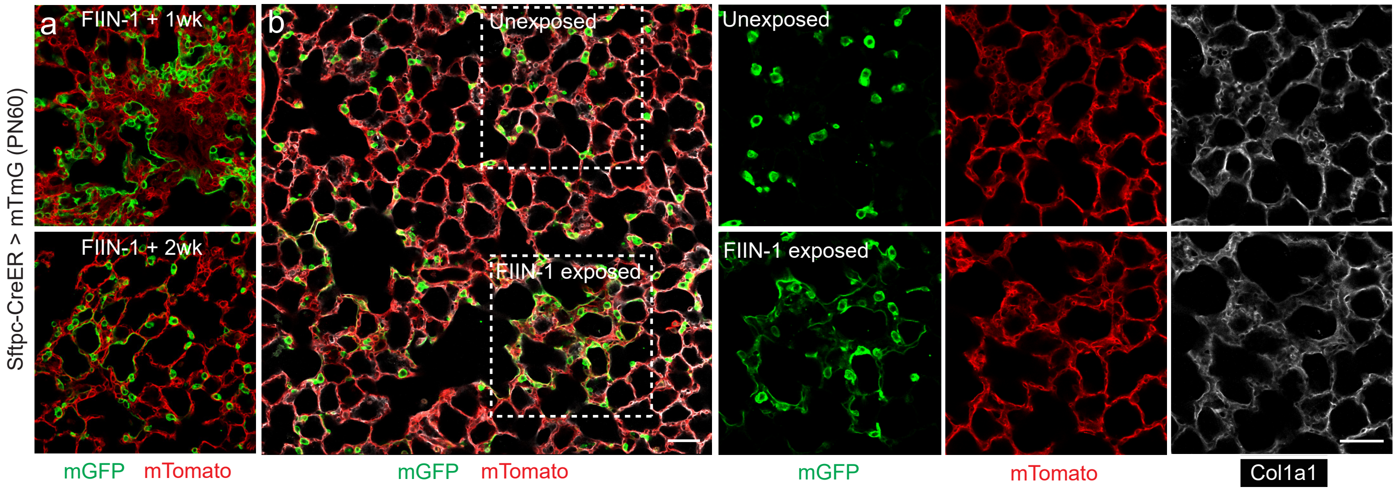

Supplementary Figure 9. Alveolar restoration following acute Fgfr inhibition. (a) Alveolar regions of adult (PN60) *Sftpc-CreER; Rosa26-mTmG* lung induced with tamoxifen to GFP label AT2 cells, then 1 week later instilled with Fgfr inhibitor FIIN-1 as in Figure 6c-f and allowed to recover for one week (upper panel) or two weeks (lower panel). Note disorganization of alveolar structure at one week that resolves by two weeks. Bar, 50 µm. (b) FIIN-1-exposed alveolar region (upper box) and control unexposed region (lower box) following FIIN-1 instillation as above and stained two weeks later for AT2 lineage marker mGFP, mTomato (marking other cell types), and collagen a1 (Col1a1). Note that the FIIN-1 exposed region has recovered and appears normal histologically with normal Col1a1 staining. Bars, 50µm. Stain repeated in biological triplicate. All experiments were repeated at least three times.

Figure	Comparison	Power
Figure 2	Control versus Fgf7, Fgf10	>0.99
Figure 4B	Fgfr2 flox versus control	>0.99
Figure 5H	Density AT2 versus Fgf+	>0.99
Figure 5P	% GFP+ AT2 cells, PN5 versus PN20	>0.99
Figure 5R	% GFP+ cells, PN5 versus PN20	>0.99
Figure 6F	fl/fl versus +/fl GFP+ AT2	>0.99
Figure 6J	fl/fl versus +/fl cCas3+	>0.99
Figure 6Q	Fgfr2iib-Fc versus veh.	>0.99
Figure 7D	EdU+ AT2 cells in SftpC-CreER	>0.99
Figure 7F	Ki67+ AT2 cells Fiin1 versus veh	>0.99

Figure	Comparison	Power
Figure 3C	Control versus Fgfr2-WT, Fgfr2-DN (Chi-Squared)	>0.99

Figure	Comparison	p-value
Figure 5I	Fgf+ vs AT2 (non-normal dist)	0.0018

Supplementary References:

1. Cohen M*, et al.* Lung single-cell signaling interaction map reveals basophil role in macrophage imprinting. *Cell* **175**, 1031-1044 e1018 (2018).

2. Treutlein B*, et al.* Reconstructing lineage hierarchies of the distal lung epithelium using single-cell RNA-seq. *Nature* **509**, 371-375 (2014).

3. Travaglini KJ*, et al.* A molecular cell atlas of the human lung from single-cell RNA sequencing. *Nature* **587**, 619-625 (2020).

4. Gillich A*, et al.* Capillary cell-type specialization in the alveolus. *Nature* **586**, 785-789 (2020).
