# Supplementary material for: Alveolar cell fate selection and lifelong maintenance of AT2 cells by FGF signaling
Source: Nat Commun. 2022 Nov 21;13:7137. doi: 10.1038/s41467-022-34059-1 (PMC9681748; doi:10.1038/s41467-022-34059-1)
Supplement: Supplementary file 2 — Reporting Summary [file 41467_2022_34059_MOESM2_ESM.pdf]

nature portfolio

Corresponding author(s): Douglas Brownfield, Tushar Desai, and Mark Krasnow

Last updated by author(s): Aug 18, 2022

Reporting Summary

Nature Portfolio wishes to improve the reproducibility of the work that we publish. This form provides structure for consistency and transparency in reporting. For further information on Nature Portfolio policies, see our Editorial Policies and the Editorial Policy Checklist.

Statistics

For all statistical analyses, confirm that the following items are present in the figure legend, table legend, main text, or Methods section.

n/a	Confirmed	
☐	☒	The exact sample size (n) for each experimental group/condition, given as a discrete number and unit of measurement
☐	☒	A statement on whether measurements were taken from distinct samples or whether the same sample was measured repeatedly
☐	☒	The statistical test(s) used AND whether they are one- or two-sided<br>*Only common tests should be described solely by name; describe more complex techniques in the Methods section.*
☒	☐	A description of all covariates tested
☐	☒	A description of any assumptions or corrections, such as tests of normality and adjustment for multiple comparisons
☐	☒	A full description of the statistical parameters including central tendency (e.g. means) or other basic estimates (e.g. regression coefficient) AND variation (e.g. standard deviation) or associated estimates of uncertainty (e.g. confidence intervals)
☐	☒	For null hypothesis testing, the test statistic (e.g. F, t, r) with confidence intervals, effect sizes, degrees of freedom and P value noted<br>*Give P values as exact values whenever suitable.*
☒	☐	For Bayesian analysis, information on the choice of priors and Markov chain Monte Carlo settings
☒	☐	For hierarchical and complex designs, identification of the appropriate level for tests and full reporting of outcomes
☒	☐	Estimates of effect sizes (e.g. Cohen's d, Pearson's r), indicating how they were calculated

Our web collection on statistics for biologists contains articles on many of the points above.

Software and code

Policy information about availability of computer code

Data collection	Zen (Carl Zeiss) were used to collect confocal images.
Data analysis	Confocal image processing and analysis was performed using freely (ImageJ 1.440 [NIH]) or commercially available software (Zen 2.3 SP1 [Carl Zeiss]). Filtering, clustering, and annotation of cells from published scRNAseq datasets was performed using R software package Seurat (version 2.3). Scripts for conducting the analysis are provided at https://github.com/dbrownfield/Brownfield_et_al .

For manuscripts utilizing custom algorithms or software that are central to the research but not yet described in published literature, software must be made available to editors and reviewers. We strongly encourage code deposition in a community repository (e.g. GitHub). See the Nature Portfolio guidelines for submitting code & software for further information.

Data

Policy information about availability of data

All manuscripts must include a data availability statement. This statement should provide the following information, where applicable:

- Accession codes, unique identifiers, or web links for publicly available datasets
- A description of any restrictions on data availability
- For clinical datasets or third party data, please ensure that the statement adheres to our policy

The scRNAseq datasets analyzed are available in the GEO repository under the accession numbers GSE109774 (Tabula Muris, https://tabula-muris.ds.czbiohub.org), GSE52583 for E18 distal lung epithelial cells, GSE109444 for adult lung mesenchymal cells, and GSE119228 for E16 lung cells. The E18 lung endothelial and mesenchymal raw sequencing data have the accession code GSE196874 .

March 2021

Field-specific reporting

Please select the one below that is the best fit for your research. If you are not sure, read the appropriate sections before making your selection.

☒ Life sciences ☐ Behavioural & social sciences ☐ Ecological, evolutionary & environmental sciences

For a reference copy of the document with all sections, see nature.com/documents/nr-reporting-summary-flat.pdf

Life sciences study design

All studies must disclose on these points even when the disclosure is negative.

Sample size	For mouse experiments, For power calculations the "Guidelines for the Care and Use of Mammals in Neuroscience and Behavioral Research" textbook was followed to determine sample size for measuring a continuous variable. Using conservative values for power (β=0.8) and significance (α=0.01), the variable C is specified (11.68). Following expected changes from prior studies (%60 change in deletion versus control) with moderate standard deviation (15%), the n calculated was 2.46, or 3 mice required for each group to reach statistical significance. Except for mouse experiments, no statistical methods were used to pre-determine sample size. As per standard practice in molecular and cell biology, we generally chose to do at least three replicates for each experiment wherever feasible.
Data exclusions	Exclusion criteria were pre-established: Cells with fewer than 500 detected genes were excluded. (A gene counts as detected if it has at least one read mapping to it). Cells with fewer than 50,000 reads (FACS) or 1000 UMI (microfluidic droplet) were excluded.
Replication	All experimental details needed to replicate the study are provided in Methods. Most experiments were performed in technical triplicate and biological triplicate. All attempts at replication were successful. We did not attempt to replicate the scRNAseq experiments due to financial constraints, however we successfully compared our scRNAseq results to those reported in Cohen et al (2018).
Randomization	When performing mouse experiments using a molecular inhibitor/activator, mice were randomly assigned to vehicle or treatment groups. For genetic experiments wherein genes were conditional deleted, groups were not random. In this case, littermates were used to normalize for age differences and multiple litters were compared to normalize for any litter-specific variances. For cell-based experiments, replicate wells were randomized as to which treatment was given.
Blinding	Images were quantified in a blinded fashion to prevent bias. For other (non-imaging) studies, investigators were blinded to group allocation during data collection and analysis .

Reporting for specific materials, systems and methods

We require information from authors about some types of materials, experimental systems and methods used in many studies. Here, indicate whether each material, system or method listed is relevant to your study. If you are not sure if a list item applies to your research, read the appropriate section before selecting a response.

Materials & experimental systems

n/a	Involved in the study
☐	☒ Antibodies
☒	☐ Eukaryotic cell lines
☒	☐ Palaeontology and archaeology
☐	☒ Animals and other organisms
☒	☐ Human research participants
☒	☐ Clinical data
☒	☐ Dual use research of concern

Methods

n/a	Involved in the study
☒	☐ ChIP-seq
☒	☐ Flow cytometry
☒	☐ MRI-based neuroimaging

Antibodies

Antibodies used	Antibodies were used at a 1:500 dilution unless otherwise noted: pro-SftpC (rabbit, Chemicon AB3786), RAGE (rat, R&D MAB1179, Clone # 175410), E-cadherin (rat, Life Technologies 13-1900, ECCD-2), Podoplanin (hamster, DSHB 8.1.1), Mucin 1 (hamster, Thermo Scientific MA5-11202 and rabbit, Novus NB120-15481), Ki67 (rat, DAKO M7249), Fgfr2 (rabbit, SCBT SC-122), cleaved Caspase 3 (rabbit, Novus NB100-56708), GFP (chicken, Abcam ab13970), Fgfr2iiib-Fc (human, R&D Systems) and Phospho-p44/42 MAPK (rabbit, CST D13.14.4E). Secondary antibodies used include Goat anti-rabbit IgG, Alexa 568 conjugated (Invitrogen, A11036); Goat anti-rat IgG, Alexa 488 conjugated (Invitrogen, A11006), Goat anti-hamster Alexa 633 (Invitrogen, A-21113), and Goat anti-Human (Invitrogen, A-11014).<br><br>For cell separation, magnetic beads conjugated to antibodies against mouse CD31 (Miltenyi Biotec, 130-097-418), CD45 (Miltenyi Biotec, 130-052-301) and Pdgfrα (Miltenyi Biotec, 130-101-547) were puchased. To positively select epithelial progenitors, a biotinylated EpCAM antibody was used (Invitrogen, 13-5791-82(clone G8.8)) with streptavidin-conjugated magnetic beads (Miltenyi Biotec, 130-048-102).

Validation	The following primary antibodies have been validated for use in mouse by the manufacturers. See the manufacturers' websites (listed below) for details. In our experiments, stainings with these antibodies were consistent with the expected patterns.<br><br>pro-SftpC (rabbit, Chemicon AB3786):https://www.emdmillipore.com/US/en/product/Anti-Prosurfactant-Protein-C-proSP-C-Antibody,MM_NF-AB3786<br>RAGE (rat, R&D MAB1179): https://www.rndsystems.com/products/mouse-rat-rage-antibody-175410_mab1179<br>E-cadherin (rat, Life Technologies ECCD-2): https://www.thermofisher.com/antibody/product/E-cadherin-Antibody-clone-ECCD-2-Monoclonal/13-1900<br>Podoplanin (hamster, DSHB 8.1.1):https://www.sigmaaldrich.com/US/en/product/mm/mabt1512<br>Mucin 1 (hamster, Thermo Scientific MA5-11202: https://www.thermofisher.com/antibody/product/MUC1-Antibody-clone-MH1-CT2-Monoclonal/MA5-11202<br>Mucin 1(rabbit, Novus NB120-15481):https://www.novusbio.com/products/muc1-antibody_nb120-15481<br>Ki67 (rat, DAKO M7249):https://www.agilent.com/en/product/immunohistochemistry/antibodies-controls/primary-antibodies/ki-67-antigen-(dako-omnis)-76239<br>Fgfr2 (rabbit, SCBT SC-122): https://www.scbt.com/p/bek-antibody-c-17<br>cleaved Caspase 3 (rabbit, Novus NB100-56708):https://www.novusbio.com/products/caspase-3-antibody-31a1067_nb100-56708<br>GFP (chicken, Abcam ab13970): https://www.abcam.com/GFP-antibody-ab13970.html?gclsrc=aw.ds|aw.ds&gclid=Cj0KCQiAxc6PBhCEARIsAH8Hff1TWts4DgkGAU7j3VqsG9wp1rS4TWi8NiAceH7YW7yXoIVZ1WZHSKgaAomcEALw_wcB<br>Fgfr2iiib-Fc (human, R&D Systems): https://www.rndsystems.com/products/recombinant-human-fgfr2-beta-iiib-fc-chimera-protein-cf_665-fr<br>Phospho-p44/42 MAPK (rabbit, CST D13.14.4E): https://www.cellsignal.com/products/primary-antibodies/phospho-p44-42-mapk-erk1-2-thr202-tyr204-d13-14-4e-xp-rabbit-mab/4370

Animals and other organisms

Policy information about studies involving animals; ARRIVE guidelines recommended for reporting animal research

Laboratory animals	1)E16 and E18 B6 male and female embryos (https://www.jax.org/jax-mice-and-services/surgical-and-preconditioning/timed-pregnant-mice).<br>2)2m old male B6, Nkx2.1-Cre (https://www.jax.org/strain/008661, Transgene) Fgfr2-flox (https://www.jax.org/strain/007569, heterozygous) bred into B6, Fgfr2-flox (homozygous) R26-mTmG (https://www.jax.org/strain/007676, homozygous).<br>3) 2m old male B6, Lyz2-Cre (https://www.jax.org/strain/004781, homozygous) Fgfr2-flox (https://www.jax.org/strain/007569, heterozygous) bred into B6, Fgfr2-flox (homozygous) R26-mTmG (https://www.jax.org/strain/007676, homozygous).<br>4) 2m old male B6, SftpC-CreER (https://www.jax.org/strain/028054, homozygous) Fgfr2-flox (https://www.jax.org/strain/007569, heterozygous) bred into B6, Fgfr2-flox (homozygous) R26-mTmG (https://www.jax.org/strain/007676, homozygous).<br>5) 2m old male B6, SftpC-CreER (https://www.jax.org/strain/028054, homozygous) bred into B6, R26-mTmG (https://www.jax.org/strain/007676, homozygous).<br>6) 2m old male B6, R26-MASTR (https://www.jax.org/strain/019013, homozygous) Fgfr2-flox (https://www.jax.org/strain/007569, heterozygous) bred into B6, Fgfr2-flox (homozygous) R26-mTmG (https://www.jax.org/strain/007676, homozygous).<br>7) Tabula Muris: C57J/B6 mice, male and female, ages 3m<br>All animals were species Mus musculus. Embryonic (E16, E18), Early postnatal (P0-P15) and adult (2-6 months, 24 months) mice were used.
Wild animals	Study did not involve wild animals.
Field-collected samples	Study did not involve field samples
Ethics oversight	All mouse experiments followed applicable regulations and guidelines and were approved by the Institutional Animal Care and Use Committee at Stanford University (Protocol 9780).

Note that full information on the approval of the study protocol must also be provided in the manuscript.
